# A Bayesian framework for modeling COVID-19 case numbers through longitudinal monitoring of SARS-CoV-2 RNA in wastewater

**DOI:** 10.1002/sim.10009

**Published:** 2024-01-14

**Authors:** Xiaotian Dai, Nicole Acosta, Xuewen Lu, Casey R. J. Hubert, Jangwoo Lee, Kevin Frankowski, Maria A. Bautista, Barbara J. Waddell, Kristine Du, Janine McCalder, Jon Meddings, Norma Ruecker, Tyler Williamson, Danielle A. Southern, Jordan Hollman, Gopal Achari, M. Cathryn Ryan, Steve E. Hrudey, Bonita E. Lee, Xiaoli Pang, Rhonda G. Clark, Michael D. Parkins, Thierry Chekouo

**Affiliations:** 1 Department of Mathematics, Illinois State University, Normal, Illinois, USA; 2 Department of Mathematics and Statistics, University of Calgary, Calgary, Alberta, Canada; 3 Department of Microbiology, Immunology and Infectious Diseases, University of Calgary, Calgary, Alberta, Canada; 4 Department of Biological Science, University of Calgary, Calgary, Alberta, Canada; 5 Advancing Canadian Water Assets, University of Calgary, Calgary, Alberta, Canada; 6 Department of Medicine, Cumming School of Medicine, University of Calgary, Calgary, Alberta, Canada; 7 Alberta Health Services, Edmonton, Alberta, Canada; 8 Water Services, City of Calgary, Calgary, Alberta, Canada; 9 Department of Community Health Sciences, University of Calgary, Calgary, Alberta, Canada; 10 Centre for Health Informatics, Cumming School of Medicine, University of Calgary, Calgary, Alberta, Canada; 11 Department of Geosciences, University of Calgary, Calgary, Alberta, Canada; 12 Department of Civil Engineering, University of Calgary, Calgary, Alberta, Canada; 13 Department of Laboratory Medicine and Pathology, University of Alberta, Edmonton, Alberta, Canada; 14 Department of Pediatrics, University of Alberta, Edmonton, Alberta, Canada; 15 Division of Biostatistics and Health Data Science, School of Public Health, University of Minnesota, Minneapolis, Minnesota, USA

**Keywords:** Bayesian negative binomial regression, Bayesian Poisson regression, functional data analysis, SARS-CoV-2, wastewater epidemiology

## Abstract

Wastewater-based surveillance has become an important tool for research groups and public health agencies investigating and monitoring the COVID-19 pandemic and other public health emergencies including other pathogens and drug abuse. While there is an emerging body of evidence exploring the possibility of predicting COVID-19 infections from wastewater signals, there remain significant challenges for statistical modeling. Longitudinal observations of viral copies in municipal wastewater can be influenced by noisy datasets and missing values with irregular and sparse samplings. We propose an integrative Bayesian framework to predict daily positive cases from weekly wastewater observations with missing values via functional data analysis techniques. In a unified procedure, the proposed analysis models severe acute respiratory syndrome coronavirus-2 RNA wastewater signals as a realization of a smooth process with error and combines the smooth process with COVID-19 cases to evaluate the prediction of positive cases. We demonstrate that the proposed framework can achieve these objectives with high predictive accuracies through simulated and observed real data.

## 1 | INTRODUCTION

Wastewater-based surveillance (WBS) has become an important tool for research groups and government agencies to investigate the pandemic caused by severe acute respiratory syndrome coronavirus-2 (SARS-CoV-2).^1^ SARS-CoV-2 genomic RNA is detectable in municipal wastewater as infected individuals shed the viruses in their feces.^2,3^ WBS has proven to be a useful tool for disease outbreak modeling and drug use monitoring before the COVID-19 pandemic^4–7^ and during the COVID-19 pandemic.^1,3,8–11^ While many countries are scaling down their resource-intensive mass clinical testing efforts to monitor the COVID-19 case burden, WBS can be easily maintained and/or adapted as an efficient and cost-effective method to assess the dynamic infections of future SARS-CoV-2 waves among monitored populations. In this article, we propose a novel statistical framework to model the relationship between WBS signals and disease infections at a population-wide level in communities served by monitored wastewater treatment plants (WWTPs). Daughton^12^ discussed the potential of WBS for estimating population-wide COVID-19 infections in communities at the beginning of the pandemic. The present study is based on original data collected in Calgary, Alberta, Canada. Compared to other studies, our study has certain advantages in modeling COVID-19 cases from WBS signals because the city has separate wastewater and stormwater collection systems, such that the latter does not influence signals derived from human feces like RNA from SARS-CoV-2 infections. The separate wastewater collection system enables effective population-wide pandemic monitoring and comparison of wastewater and clinical data.^1^

In this study, we aim to predict new positive cases reported (or test positivity rate) using the abundance of genomic RNA copies in city wastewater. The RNA copies and infectious virus of SARS-CoV-2 tend to have some commonalities and differences in terms of shedding patterns as summarized in Puhach et al^13^ as follows: (i) most of the viral load and shedding of both genomic RNA copies and infectious virus copies happens around the peak of symptoms in COVID-19 patients, especially during the first 5 days of the onset of symptoms^13^; (ii) both genomic RNA copies and infectious virus copies decline over the course of an infection in the viral load, reaching low or undetectable levels two weeks after the onset of symptoms^14,15^; (iii) overall patterns of viral shedding dynamics are very similar across SARS-CoV-2 variants, but variants of concern (eg, Alpha, Beta, Gamma, Delta, and Omicron) display some differences in viral loads,^16,17^ which is important for our study as the prediction model built should be somewhat robust to different variants; (iv) similar genomic RNA viral loads can be detected in vaccinated and unvaccinated patients, while vaccination can significantly reduce infectious virus loads.^18,19^ We measure genomic RNA copies in our study, which makes our measurements less sensitive to vaccination status or variants.

A significant proportion of the available COVID-19-related literature has dedicated its effort to modeling the association between disease incidence and/or prevalence and wastewater observations. There are existing Bayesian methods to predict or associate positive cases with the abundance of SARS-CoV-2 RNA in wastewater. For example, Peccia et al^9^ proposed a Bayesian regression model to predict daily COVID-19 positive cases using WBS signals. However, the method is not directly applicable to the Calgary study or many other wastewater studies as it is common that WBS samplings and observations are intermittent and even sparse depending on available resources. Hoffmann and Alsing^20^ developed Bayesian hierarchical models to predict RNA concentration from a small sample of COVID-19 patients. However, we focus on community-wide case prediction using large-scale wastewater data with more confounding factors. Besides the Bayesian methods, many other proposed methods can be classified as a susceptible-infected-removed (SIR) model, which is commonly used to analyze the transmission patterns of different infectious diseases. For example, McMahan et al^21^ proposed to use a system of differential equations to predict the number of infected individuals based on the mass rate of SARS-CoV-2 RNA in wastewater. However, McMahan et al did not discuss handling sparse WBS signals either. Compared to the system of differential equations, our approach uses functional regression to predict the number of positive cases as well as functional data analysis to estimate the dynamic of WBS signals, which imputes sparse WBS signals and enables straightforward statistical and clinical interpretations. Also, we aim to estimate unknown parameters directly from the observed data instead of imposing potentially limited information on the predictive model. Roberto Telles et al^22^ pointed out that SARS-COV-2 dissemination patterns can be heavily influenced by weather conditions or even government policies, which makes the SIR assumptions of susceptibility, immunity, and spreading patterns differ greatly around the globe. Our proposed framework serves as an alternative to the SIR model and requires little prior knowledge about the model parameters. Another family of proposed methods uses various regression and machine learning methods to predict COVID-19 cases. For example, Koureas et al^23^ used the random forest algorithm to predict COVID-19 cases in two Greek cities using wastewater RNA. Morvan et al^24^ used gradient-boosted regression trees to infer infection prevalence in major cities of England using SARS-CoV-2 RNA in wastewater. Galani et al^25^ proposed to predict COVID-19-related hospitalizations from leading wastewater and pandemic health indicators using linear and nonlinear regressions including neural networks. Again, the handling of irregular and sparse WBS signals is not considered in these proposed methods. Compared to conventional regression models, our proposed framework is based on a generalized functional regression model with time-dependent wastewater signals and a time-dependent random “frailty” term accounting for self-selection bias in observed cases, as the wastewater and clinical data have a temporal structure instead of being independent observations. To model the clinical outcome as a function of time, our proposed framework can incorporate the mean and covariance structure of irregular and sparse daily observations. The proposed framework also estimates the underlying WBS function jointly with the functional regression, so that the estimated WBS function parameters are also conditional on the clinical outcome.

Overall, there are two main challenges facing the statistical modeling of the relationship between the longitudinal and multicenter observations of viral copies in municipal wastewater and daily reported positive cases. The first challenge pertains to the frequency of WBS observations. In Calgary, the number of viral copies in municipal wastewater is estimated by taking wastewater samples from different centralized WWTPs and quantifying viral RNA using the reverse transcription-quantitative polymerase chain reaction (RT-qPCR) analysis.^1^ Measuring viral gene abundance in wastewater samples induces variability introduced by unavoidable technical and statistical errors or interference. Furthermore, wastewater samples often cannot be collected from WWTPs every day or not always regularly, which means the WBS observations can be irregular and sparse.^26^ The proposed statistical framework smooths and interpolates the trajectory of WBS signals so that they can be used as the independent variable of the regression predicting the number of positive cases. The smoothed and interpolated trajectory of WBS signals should also reflect the true dynamic of viral copies in municipal wastewater out of noisy and sparse observations and can be used as a reliable indicator of a disease outbreak in the region serviced by municipal WWTPs when there is a significant increase in WBS signal. The second main challenge arises from the clinically reported positive cases. This depends on the policy and capacity a jurisdiction has for mass testing (ie, the number of people tested and the resources to test them promptly). The proposed framework accounts for the uncertainty in predicting the number of reported cases. Moreover, testing results can lag the pandemic’s progression, because testing is usually prompted by symptoms^9,27^); in other words, patients typically start shedding viruses and contributing to the wastewater signal before getting tested. Therefore, the proposed framework predicts the positive cases as a function of time from WBS signals as leading indicators.

Acosta et al^1^ analyzed the correlation between wastewater viral data and clinical cases in the city of Calgary, but did not study the prediction of clinical cases and its uncertainty. In this article, the proposed framework uses generalized Bayesian functional regression with Poisson-distributed or negative binomial (NB)-distributed response to predict the time-dependent positive case counts from the longitudinal observations of viral copies in wastewater samples (ie, WBS signals). The longitudinal WBS signals are summarized into high-quality predictors using a functional data analysis method called the functional principal component analysis (FPCA).^28–31^ FPCA can detect the major trends in the longitudinal observations of viral gene abundance while removing small fluctuations that are likely to be caused by technical errors or interference. The FPCA process is incorporated into the Bayesian functional regression, and the unknown parameters in the regression and FPCA process are estimated together in a joint framework. We demonstrate that the proposed framework can successfully detect true WBS signals from noisy and irregular observations, and accurately predict the number of reported positive cases and positivity rates from WBS signals through various simulated data. We applied the proposed framework to the Calgary (Alberta, Canada) SARS-CoV-2 data, an original WBS monitoring dataset collected by the WBS team at the University of Calgary during the COVID-19 pandemic. This dataset provides as a proof-of-concept study for the performances of the proposed framework. The proposed framework should also have a broader impact on future epidemiological studies.

In Section2, we introduce the proposed framework’s data collection process and the technical details (ie, model framework, prior setup, and algorithm). In Section 3, we evaluate the performance of the proposed framework on simulated datasets and compare its performance to a two-stage frequentist method. We also apply the proposed framework to the original data collected in Calgary. In Section 4, we summarize the findings and discuss the contributions of the proposed framework.

## 2 | METHODS

### 2.1 | Overview of the Calgary wastewater study

**FIGURE 1:**
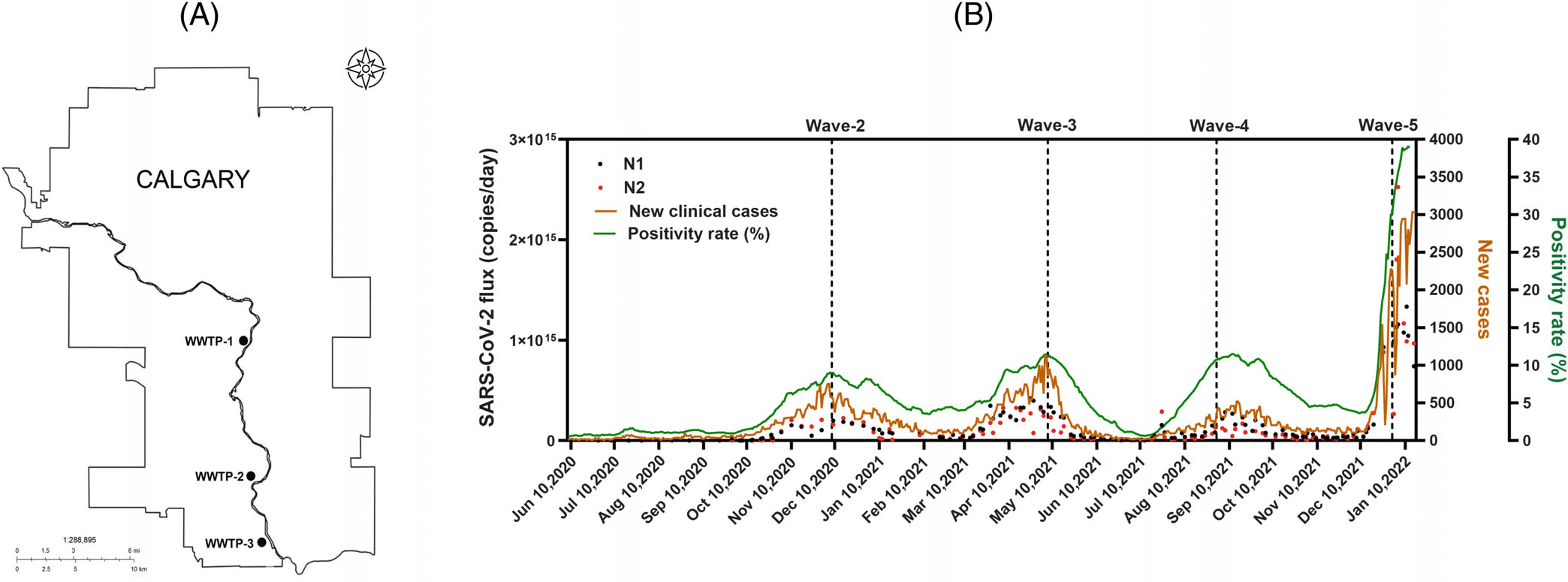
(A). The map of Calgary City, Canada. There are three WWTPs labeled as WWTP-1, WWTP-2, and WWTP-3. (B). The daily new positive cases in the city of Calgary are shown in the brown curve. The daily positivity rate (the number of positive cases divided by the number of people tested) is shown in the green curve. The total number of copies of N1 and N2 in Calgary wastewater are shown in black and red dots, respectively.

Wastewater samples used for this study were collected from June 2020 to June 2022 at each of Calgary’s three wastewater treatment plants (WWTPs) (Figure 1A). The three plants serve an estimated population of 1 441 268 people in Calgary and surrounding communities.^1^ Composite raw wastewater samples were collected from each of the three WWTPs for up to three times per week. WWTP wastewater samples were collected by the City of Calgary Water Utility Services staff and/or the University of Calgary research team using a consistent standard protocol. Wastewater samples were transported to the University of Calgary’s Advancing Canadian Water Assets (ACWA) lab for preprocessing and RNA isolation immediately after collection. Nucleic acid extraction from wastewater was performed at the ACWA lab using the Sewage, Salt, Silica, andSARS-CoV-2 (4S) protocol.^27,32^ Extracted nucleic acids were transported on dry ice to the Health Sciences Center at the University of Calgary for subsequent reverse transcription-quantitative polymerase chain reaction (RT-qPCR) analysis. This project was approved by the Conjoint Regional Health Ethics Board (REB20–1252). See Acosta et al^1^ for technical details of the wastewater sample collection and storage process.

For the subsequent RT-qPCR analysis, United States Center for Disease Control (US-CDC) primers and probes were used to amplify two regions of the nucleocapsid gene (ie, N1 and N2). Total mass flux of SARS-CoV-2 RNA concentration measured as copies per day for the City of Calgary was calculated, in which the daily flow rate of each WWTP was multiplied by copies/mL of SARS-CoV-2 RNA measured in wastewater to generate the number of copies per day for each WWTP. For this reason, the RNA concentration (copies per day) across three WWTPs in Calgary can be aggregated into one data point per sampling day to represent the city-wide SARS-CoV-2 burden. The numbers of copies per day for N1 and N2 are shown in Figure 1B, and the values are log-transformed for further statistical modeling. See Section A of the Supplementary Information for more details about the RT-qPCR analysis.

All health services and COVID-19 diagnostic testing in Calgary (Alberta, Canada) and surrounding communities are performed through the publicly funded Alberta Health Services (AHS). Throughout the study period, daily positive cases tested and diagnosed by the AHS, and test positivity rates (ie, daily percentage of positive cases based on the total amount of tests performed in the province of Alberta) were collected and recorded. The COVID-19 testing in the city of Calgary is voluntary except for a few high-risk neighborhoods such as hospitals. The AHS also reports accurate vaccination data in Alberta as all the government-approved COVID-19 vaccines are administered through the universal AHS health insurance plan.

### 2.2 | Normalization of wastewater signals

We first normalize the wastewater raw observations to account for seasonality and weather patterns (eg, temperature, water flow volume) before using them to predict new positive cases reported. The normalization process is performed using a regression model:

$${\tilde{W}}_{k}\;=\;S\beta^{\left(k\right)}+W_{k},$$

where ${\tilde{W}}_{k}$ is a vector of the raw wastewater observations for the kth target gene, S is a matrix containing seasonality and other weather indicators recorded (eg, daily average temperature and daily total wastewater flow) at the timestamps of the raw wastewater observations, and $\beta^{\left(k\right)}$ are the regression coefficients estimated via ordinary least-squares, and obtained from the training period. For example, to model seasonality, we can include four dummy variables in the matrix S to represent the season status of an observation date. The same ordinary least-square estimates of the regression coefficients are applied to samples in the prediction period so that the adjustment factors are consistent in the training and testing data. $W_{k}$ is the vector of residuals and will be used as the wastewater signals after adjusting for seasonality and other weather pattern variables. The normalization process is more important in jurisdictions without separate wastewater and stormwater systems, where weather patterns and water flow volumes can have a profound impact on wastewater collections and WBS measurements. After normalization, the WBS measurements can be comparable under different climate conditions and wastewater systems.

In this study, the S matrix contains three main covariates: the four season dummy variables, the daily average temperature recorded near the Calgary International Airport (Latitude: 51°07′21″ N; Longitude: 114°00′48″ W), and the daily total wastewater flow (in one thousand $m^{3}$). Two multiple regressions are fitted for N1 and N2 genes on training data in 2020 to obtain $W_{k}$, and the estimated regression coefficients are used to normalize raw observations in 2021 (ie, get $W_{k}$ on the test set). In both N1 and N2 regressions, all regression coefficients in $\beta^{\left(k\right)}$ are highly significant (with *P*-values < 0.0001). The wastewater observations of N1 and N2 genes before and after normalization are shown in Figure S13 of the Supplementary Information. After normalization, the trends of pandemic waves can still be visualized in $W_{k}$, which would be important for predicting new positive cases.

### 2.3 | Statistical framework

The proposed framework aims to model and predict positive case counts released by local authorities (eg, Alberta Health Services) from WBS signals. The WBS signals are produced from longitudinal and multicenter samplings and measurements, whose observations can be irregular and sparse. The daily city-wide WBS signal is aggregated by averaging the total number of viral copies at multiple WWTPs across Calgary. WBS signals are considered potential leading indicators of the number of new positive cases. They are used as covariates in a lagged functional regression model with Poisson-distributed or NB-distributed outcomes (ie, case numbers). This analysis is carried out in a Bayesian framework, allowing us to characterize multiple sources of uncertainty straightforwardly when estimating the lagged associations of interest. In the proposed framework, we use the observed wastewater signals to estimate the underlying, unobserved trajectory of true viral copies in the WWTP samples. We then model the association between the underlying trajectory of viral copies in wastewater at a lagged timestamp and the number of positive cases through a generalized functional regression model. All the unknown parameters in the proposed Bayesian framework are sampled and estimated in a joint framework using a Markov chain Monte Carlo (MCMC) algorithm.^33,34^

Let Y(t) be the daily reported case number on the tth day. We use two distributions to model Y(t). We first model Y(t) as a Poisson distribution:

(1) Y(t)∣λ(t)~Poisson(λ(t)),

where λ(t) is the mean parameter of the Poisson distribution and can be seen as a latent variable that represents the expectation of daily case counts at time t. Although Poisson modeling is widely used in count data analysis, it requires that the mean and variance be equal which limits its use for overdispersed data. To overcome such a situation, we also model Y(t) as a NB distribution:

(2) $$Y\left(t\right)|\lambda \left(t\right)\;\sim 𝒩ℬ\left(\lambda \left(t\right),\eta \right),$$

where λ(t) is the expectation of the NB distribution and η is a hyperparameter controlling the dispersion of Y(t)’s distribution. The variance of Y(t) is $\lambda (t)+\lambda (t{)}^{2}/\eta$ which is larger than λ(t).

For both distributions, the latent expectation λ(t) at time t is modeled as

(3) $$\begin{array}{l} \text{log}\left(\lambda \left(t\right)\right)\;=\;\text{log}\;\left(E\left[Y\left(t\right)\right]\right)\;=\;\text{log}\left(O\left(t\right)\right)+\phi \left(t\right)\\ \;+\beta_{0}+\sum_{p=1}^{P}\beta_{p}{\left[\hat{X}\left(t-\delta \right)\right]}^{p}+\sum_{q=1}^{Q}\beta_{q}^{c}\left[C_{q}\left(t-\delta_{q}\right)\right], \end{array}$$

where

- λ(t) is a latent and “intermediate” parameter introduced for ease and clarity of presentation. It is fixed conditional on the other parameters in Equation (3). Given $\lambda \left(t_{1}\right)$ and $\lambda \left(t_{2}\right)$ for $t_{1}\neq t_{2},$ $Y\left(t_{1}\right)$ and $Y\left(t_{2}\right)$ are independent.
- O(t) is the offset of the regression and represents the number of people tested (ie, Y(t)∕O(t)=positivityrate). It is observed and fixed.
- ϕ(t) is the random effect for the bias in the number of reported cases compared to the actual number of new cases, which is also referred to as the “frailty” term. The motivation behind the “frailty” term is that the COVID-19 mass testing is completely voluntary in Calgary and many other communities, so there exists a self-selection bias in the residents being tested and the number of positive cases reported by the local authorities. This bias exists not only in the pandemic of COVID-19, but also in the transmission data of other infectious diseases. We introduce a time-dependent random effect term,^35^ ϕ(t), to account for the bias. ϕ(t) has a normal prior with mean depending on $\phi (t-1):\phi (t)\;\sim 𝒩(\alpha \phi (t-1),\sigma_{\phi }^{2})$, so ϕ(t) is an autoregressive process of order 1 (an AR(1)) involving two unknown parameters: α∈(−1,1) and $\sigma_{\phi }^{2}>0$. The first frailty ϕ(1) follows $𝒩(0,{\;\sigma }_{\phi }^{2})$. ϕ(t) can also account for the proportion of variation in the response variable that can only be explained by missing predictor(s) such as unavailable community demographic data.
- $\overset{ˆ}{X}(t-\delta )$ is the estimated RNA wastewater signal function at time t−δ (ie, the mean across target genes of the smoothed RNA concentration) obtained from WBS observations. $\overset{ˆ}{X}(t-\delta )$ is a fixed parameter conditional on $\mu \left(t\right),$ ψ(t), and $\xi_{l}$, which are introduced later in Section 2.3.1. In the term $[\overset{ˆ}{X}(t-\delta ){]}^{p},$ δ is the time lag, which means the model predicts daily positive cases from WBS observations collected δ days ago. The underlying assumption is that WBS signals should serve as a leading indicator of new positive cases. For example, if public health researchers or administrators aim to predict the positive cases from wastewater samples collected a week ago, a typical choice of δ would be seven. According to Puhach et al,^13^ the viral loads in most infected individuals reach low or undetectable levels 2 weeks after the onset of symptoms. That means a δ value larger than 14 would not make much sense in terms of biological interpretation. In addition, Morvan et al^24^ pointed out that SARS-CoV-2 signals in wastewater appear at least 5 days earlier in comparison to clinical testing data. Therefore, the choice of δ value should mostly depend on its biological meanings. The power p in the term $[\overset{ˆ}{X}(t-\delta ){]}^{p}$ for p=1,…,P enables higher-order wastewater signal terms, which can be useful when there is a clear indication that the relationship between wastewater signal and clinical data is not linear (when $P>1).\;\beta_{p}$ is the regression coefficient of $[\overset{ˆ}{X}(t-\delta ){]}^{p}$. Within the biologically meaningful and relatively small range of δ values, the mean squared prediction error (MSPE) on test data can be used to select δ and/or P.
- In addition to the WBS observations, other fixed clinical covariates that can be considered leading indicators of newly reported cases are also included as $C_{q}\left(t-\delta_{q}\right)$, with their random regression coefficient $\beta_{q}^{c}$. In our study, the vaccination rates of Alberta residents (ie, the percentage of the population who have received at least one dose of government-approved COVID-19 vaccines) are used as a clinical covariate. $\delta_{q}$ represents the time lag of the effect of the covariate(s) on the new cases.

#### 2.3.1 | RNA wastewater signal function

Let $W_{tk}$ be the observed discrete wastewater genomic RNA abundance for the kth gene target (eg, N1, N2, or other genes) on the tth day. The observed WBS signal can be decomposed as:

(4) $$W_{tk}\;=\;\mu \left(t\right)+\sum_{l=1}^{\infty }\xi_{kl}\psi_{l}\left(t\right)+\epsilon_{tk},$$

and the underlying signal function X(t) is unobserved but estimated as a smoothed average of $W_{tk}$’s for all k:

(5) $$\hat{X}\left(t\right)\;=\;\mu \left(t\right)+\sum_{l=1}^{L}\xi_{l}\psi_{l}\left(t\right),$$

where k=1,…,K, μ(t) is a smoothed mean function of the WBS signal functions, $\psi_{l}$’s are eigenfunctions generated from the functional principal component analysis (FPCA) process.^30^ μ(t) and $\psi_{l}(t)$’s are derived and fixed given observed $W_{tk}$’s, while $\xi_{l}$’s are random coefficients. The coefficients of eigenfunctions in Equation (5) are defined as $\xi_{l}\;=\;\frac{1}{K}\sum_{k=1}^{K}\;\xi_{kl}$, where $\xi_{kl}$’s are the gene target and eigenfunction-specific scores. We only need to average $\xi_{kl}$’s because all the K target genes (ie, N1 and N2) share the same μ(t) and $\psi_{l}(t)$. The goal is to remove the error component $\epsilon_{tk}$ and to obtain the estimate $\overset{ˆ}{X}(t)$ as detailed in Equation (5). Note that the summation from 1 to ∞ in Equation (4) indicates that functional data are infinite-dimensional. We only keep the first L eigenfunctions such that the first L eigenfunctions can explain at least 99% of the total variation in the observed data when estimating $\overset{ˆ}{X}(t)$. To estimate μ(t) and $\psi_{l}$’s, the observed signals $W_{tk}$’s for all K gene targets are pooled together to estimate the sample mean function and sample covariance matrix of $W_{tk}$’s. The estimated mean function μ(t) is obtained by smoothing the sample mean function. The smoothed covariance surface is discretized to estimate the eigenfunctions $\psi_{l}$’s as detailed in Rice and Silverman.^36^ The dimensions of the sample covariance matrix depend on the number of distinct timestamps in $W_{tk}$ observations. As the sample covariance matrix is noisy, Yao et a^30^ employed the local linear smoothers^37^ for function and surface estimation (ie, covariance matrix can be seen as a bivariate surface). $\xi_{kl}$’s (for all k) have a mean of zero and variance of $\Lambda_{l}$, where $\Lambda_{l}$ is the eigenvalue associated with eigenvector $\psi_{l}(t)$. Here we treat the average of $\xi_{kl}$’s for the lth eigenvector, $\xi_{l}$, as one single random parameter and estimate $\xi_{l}$ using the empirical estimates of $\Lambda_{l}$ as implemented in the R package *refund*.^38^ After estimating μ(t), $\xi_{l}$ and $\psi_{l}(t)$, we can reconstruct $\overset{ˆ}{X}(t)$ using Equation (5).

#### 2.3.2 | Prior distributions

The prior distribution for the score $\xi_{l}$ is

$$\xi_{l}\;\sim N\left(0,\frac{\Lambda_{l}}{K}\right),$$

where $\Lambda_{l}$ is the eigenvalue associated with the lth eigenfunction $\psi_{l}(t)$ and the variance of $\xi_{kl}$. As $\xi_{l}$ is the average of the independent variables $\xi_{kl}$’s (k=1,…,K), the variance of $\xi_{l}$ is $\frac{\Lambda_{l}}{K}$. The prior distributions imposed on the random intercept and the random regression coefficient in Equation (3) are

$$\beta_{0}\;\sim N\left(0,100^{2}\right)\;\text{and}\;\beta_{p}\;\sim N\left(0,\sigma_{\beta }^{2}\right),$$

where $\beta_{0}$ follows a normal prior with a large variance, which is essentially a flat and non-informative prior. $\beta_{p}$’s follow a normal prior with zero mean and variance $\sigma_{\beta }^{2}$, where $\sigma_{\beta }^{2}$ is also a random parameter. $\sigma_{\beta }^{2}\;\sim inverseGamma(0.1,\;0.1)$ follows an inverse Gamma prior distribution where the shape and scale parameters are both 0.1, which makes the prior non-informative. The non-informative priors are used because we assume that little prior knowledge of the parameters is available and the parameters are mostly estimated from the observed data. The coefficient of the AR⁡(1) process in ϕ(t)’s prior follows α~𝒰(−1,1), a uniform distribution from −1 to 1. That means we assume the process of ϕ(t) is weak-sense stationary, but we do not impose additional prior information on α. Again, the variance parameter $\sigma_{\phi }^{2}\;\sim inverseGamma(0.1,\;0.1)$ follows an inverse Gamma distribution.

### 2.4 | MCMC algorithm and posterior sampling

In this joint Bayesian framework (ie, Equations(1) and (5) are estimated jointly), all unknown parameters are sampled and estimated in a joint MCMC algorithm using the *RStan* package^34^ in R, with which we can specify the prior distributions and likelihood, establish the regression model, and run MCMC diagnostics. The joint posterior distribution of unknown parameters is given by

$$\begin{array}{l} \;p\left(\beta_{0},\beta ,\beta^{c},\alpha ,\sigma_{\phi }^{2},\phi \left(.\right),\xi ,\sigma_{\epsilon }^{2}|Y\left(.\right),{\left\{W_{.k}\right\}}_{k\;=\;1}^{K}\right)\\ \propto p\left(Y\left(.\right),{\left\{W_{.k}\right\}}_{k\;=\;1}^{K}|\beta_{0},\beta ,\beta^{c},\alpha ,\sigma_{\phi }^{2},\phi \left(.\right),\xi ,\sigma_{\epsilon }^{2}\right)\\ \;\times \left(\prod_{t=2}^{T}p\left(\phi \left(t\right)|\phi \left(t-1\right),\alpha ,\sigma_{\phi }^{2}\right)\right)p\left(\beta_{0}\right)p\left(\beta \right)p\left(\beta^{c}\right)p\left(\xi \right)p\left(\sigma_{\epsilon }^{2}\right)p\left(\alpha \right)p\left(\sigma_{\phi }^{2}\right), \end{array}$$

where $\beta \;=\;\left[\beta_{1},\ldots ,\beta_{P}\right]$ and $\xi \;=\;\left[\xi_{1},\ldots ,\xi_{L}\right]$. Note that the latent parameter λ(t) is fixed conditional on $\beta_{0},\beta ,\beta^{c},\overset{ˆ}{X}(t-\delta )$, and $\phi \left(t\right),\overset{ˆ}{X}(t-\delta )$ is fixed conditional on $\xi_{l}$’s, μ(t−δ), and ψ(t−δ), and hence they are not included as a random parameter here. The RStan package uses the Hamiltonian Monte Carlo algorithm with the No-U-Turn Sampler^39^ to sample unknown parameters from the joint posterior distributions.

The regression coefficients and residual terms in the functional regression and the random scores related to the FPCA process are updated iteratively. The model-fitting algorithm of the proposed framework can be described as:

1. Find the value of L. Also, find the first L eigenfunctions $\psi_{l}$’s and their eigenvalues ${ℰ}_{l}$’s. Fit the model with multiple values of P. Determine the optimal choice of P by performing residual diagnostics and numerical assessments such as mean squared prediction error on predicted case numbers.
2. Update unknown parameters $\xi_{kl}$’s, $\beta_{0},$ $\;\beta_{p}$’s, $\beta_{p}^{c}$’s $\sigma_{\beta }^{2},\;\alpha$, and $\sigma_{\phi }^{2}$ iteratively, conditional on the observed case numbers and generated eigenvectors in step 1. The observations used to fit the model come from the training period only: Y(t) and $W_{tk}$’s where $1\leq t\leq N_{\text{train}}$, and $N_{\text{train}}$ is the number of days in the training period.

Note that the model is fitted without any knowledge from the prediction period (ie, the test period). After the model is fitted, the prediction algorithm can be summarized as:

1. For the prediction period $t>N_{\text{train}}$, the estimated mean $\overset{ˆ}{\lambda }(t)$ of response Y(t) for new data point can be derived as
(6) $$\hat{\lambda }\left(t\right)\;=\;O\left(t\right)\;\text{exp}\;\left\{\hat{\phi }\left(t\right)\;+\;{\hat{\beta }}_{0}\;+\;\sum_{p=1}^{P}{\hat{\beta }}_{p}{\left[\hat{X}\left(t\;-\;\delta \right)\right]}^{p}\;+\;\sum_{q=1}^{Q}{\hat{\beta }}_{q}^{c}\left[C_{q}\left(t\;-\;\delta_{q}\right)\right]\right\},$$
  with the random frailty $\overset{ˆ}{\phi }(t)$ sampled from $𝒩(\overset{ˆ}{\alpha }\overset{ˆ}{\phi }(t-1),{\overset{ˆ}{\sigma }}_{\phi }^{2})$, where ${\overset{ˆ}{\beta }}_{0},$ ${\overset{ˆ}{\beta }}_{p}$’s, ${\overset{ˆ}{\beta }}_{q}^{c}$’s, $\overset{ˆ}{\alpha }$, and ${\overset{ˆ}{\sigma }}_{\phi }^{2}$ are after-burn-in posterior samples. If O(t) is unknown in the prediction period, we can set O(t)≡1 for $t>N_{\text{train}}$, and the positivity rate is predicted.
2. $\overset{ˆ}{X}(t-\delta )$ also needs to be extended into the prediction period. A straightforward approach is to combine the wastewater observations in both training period and prediction period to re-generate the mean function μ(t) and eigenfunctions $\psi_{l}\left(t\right)$’s, which naturally extends the mean function and eigenfunctions into prediction period. $\overset{ˆ}{X}(t-\delta )$ can be calculated as
$$\hat{X}\left(t-\delta \right)\;=\;\mu \left(t-\delta \right)+\sum_{l=1}^{L}{\hat{\xi }}_{l}\psi_{l}\left(t-\delta \right),$$
  where ${\overset{ˆ}{\xi }}_{l}$ is an after-burn-in posterior mean.
3. The last step is to sample Y(t) for $t>N_{\text{train}}$. Note that in the training period, Y(t) is observed and fixed. In the prediction period, Y(t) can be estimated as $\overset{ˆ}{Y}(t)\;=\;\overset{ˆ}{\lambda }(t)$, where $\overset{ˆ}{\lambda }(t)$ can be seen as the after-burn-in posterior samples of the expectation of $\overset{ˆ}{Y}(t)$. The posterior mean and variance of $\overset{ˆ}{Y}(t)$ are used to assess prediction performance.

The performances of the proposed framework are measured by how well new positive cases can be predicted from WBS signals and how well the framework can detect true signal function from noisy WBS observations. Especially, for the simulated data, the true WBS signal function is known before adding artificial noise.

## 3 | RESULTS

### 3.1 | Simulations

#### 3.1.1 | Simulation designs

Here we use simulated data to demonstrate the effectiveness of the proposed framework for predicting new positive cases from WBS signals. We mimic the real data in the Calgary study to assess the performance of the proposed framework and other methods. In all simulation designs, the predictive models are trained on simulated WBS observations and positive cases from June 2020 to December 2020 (ie, training period) and are used to predict on simulated data for the subsequent 12-month period from January 2021 to December 2021 (ie, prediction period). As mentioned above, the predictive models are trained without knowledge from the prediction period (or test period).

For each design, noise is added to simulated WBS signals and the true signal function is unknown to the proposed framework. We are interested in how well the framework can model clinical outcomes using WBS signals and how well the framework can detect true signal function from noisy observations. We run 50 000 MCMC iterations with 25 000 burn-in iterations for each fitting of the proposed framework. Therefore, the 25 000 after-burn-in MCMC samples are used to assess the predictions of simulated positive cases. We implement five simulation designs:

- **Simulation design 1**: In the first simulation design, we let L=2, and the observed number of copies for N1 is generated as $W_{1}(t)\;=\;\mu (t)+\xi_{11}\psi_{1}(t)+\xi_{12}\psi_{2}(t)+\epsilon_{t1}$ and that for N2 is generated as $W_{2}(t)\;=\;\mu (t)+\xi_{21}\psi_{1}(t)+\xi_{22}\psi_{2}(t)+\epsilon_{t2}$, where μ(t), $\psi_{1}(t)$, and $\psi_{2}(t)$ are generated from the Calgary wastewater history, and $\epsilon_{tk}\;\sim N(0,\;1)$. The random scores are generated from normal distributions: $\xi_{11}\;\sim N\left(0.3,0.05^{2}\right),\xi_{21}\;\sim N\left(-0.3,\;0.05^{2}\right),\xi_{12}\;\sim N\left(0.05,{\;0.01}^{2}\right)$, and $\xi_{22}\;\sim N\left(-0.05,0.01^{2}\right)$. Regarding the clinical outcome, the number of positive cases is generated as Y(t)~Poisson(λ(t)), and the mean parameter for the number of positive cases is generated as $\lambda (t)\;=\;exp(log(O(t))+\beta_{0}+0.5X(t-7)+\phi (t))$. The offset term O(t) is randomly sampled from integers between 450 and 500 and the intercept term $\beta_{0}$ is set be −1.5. The residual term ϕ(t) is generated as ϕ(t)~N(0.05ϕ(t−1),0.01) and ϕ(1)~N(0,0.01).
- **Simulation design 2**: In the second simulation design, most settings are unchanged relative to Simulation 1 except for the highest power of the WBS function P increased to 2. The mean parameter for the number of positive cases is generated as $\lambda (t)\;=\;exp\left(log(O(t))+\beta_{0}+0.5X(t-7)+0.1[X(t-7){]}^{2}+\phi (t)\right)$. This design aims to test the robustness of the proposed framework when the effect of wastewater on positive cases is not linear.
- **Simulation design 3**: In the third simulation design, one-tenth of the actual daily number of people tested in Calgary data is used as the offset term, which means the offset is not simulated in this simulation. For other parameters, the settings are the same as those in Simulation 1.
- **Simulationdesign4**: In the fourth simulation design, the observed WBS signals are irregular and sparse. We have 20% of the simulated N1 and N2 copies that are randomly chosen to be missing values. All the other settings are the same as those in Simulation 1. This design aims to test the robustness of the proposed framework when the longitudinal observations of WBS signals contain many missing values.
- **Simulation design 5**: This design is motivated by the real-life scenario that the offset term (ie, the total number of people tested) is unknown in the prediction period. All the settings in Simulation 5 are the same as those in Simulation 1. In this design, the offset term in the training period is randomly sampled from integers between 450 and 500. However, the offset term is known when fitting the regression model (ie, known in the training period), while the offset is assumed to be unknown when making predictions (ie, unknown in the prediction period). This design aims to predict the positivity rate instead of the case count. The mean parameter for the number of positive cases is generated as $\lambda (t)\;=\;exp\left(log(O(t))+\beta_{0}+0.5X(t-7)+\phi (t)\right)$. The prediction algorithm remains the same as detailed in Section 2.4 while setting the offset O(t)=1.
- **Simulation design 6**: In the last simulation design, the number of positive cases is generated as Y(t)~𝒩ℬ(λ(t),100), where the NB distribution has a mean of λ(t) and a variance of $\lambda (t)+\lambda (t{)}^{2}/100$. All the other settings are the same as those in Simulation 1.

#### 3.1.2 | Simulation results

We compare the simulated numbers of new positive cases in the prediction period with the predicted number of positive cases to calculate the mean squared prediction error (MSPE) ${∥Y_{T\times 1}-{\overset{ˆ}{Y}}_{T\times 1}∥}_{2}^{2}/T$, where $∥.{∥}_{2}$ denotes the $l_{2}$ norm, T is the number of days in the prediction period, and $\overset{ˆ}{Y}$ is a T×1 vector containing posterior means of $\overset{ˆ}{Y}(t)$ to assess the accuracy of predictions. Therefore, we calculate the average of the MSPEs as well as the standard error of the MSPEs. The MSPEs for all simulation designs are presented in Table 1. We fit the proposed framework with both P=1 and P=2 to justify the optimal choice of P. The comparison results of P=1 vs P=2 can be found in Supplementary Information Section B. We also compare the true WBS signal function with the estimated WBS signal function to calculate the mean squared error (MSE) ${\left\|X-\hat{X}\right\|}_{2}^{2}/T$ for WBS signal function estimation. The numerical results are also included in the Supplementary Information Section B.

We also compare the proposed unified framework with two-stage frequentist methods, which first impute the missing values in WBS signals using the FPCA method and then predict the positive cases from the imputed and smoothed WBS signals using a Poisson regression. The Poisson regression is implemented by the *glm* function in R. We denote this method by *FPCA*+*Poisson*. However, the conventional generalized linear regressions such as the Poisson regression assume the independence of samples and do not consider time-dependency among residuals like the proposed framework. Therefore, we also compare the autoregressive integrated moving average (ARIMA),^40^ a time-series forecasting method, with our framework here. The ARIMA model is implemented by the *auto.arima* function in the R package *forecast*,^41^ where the autoregressive order and moving average order are optimized automatically. The ARIMA model also uses the FPCA-imputed WBS signal as a predictor. Also, the count data (case numbers) are log-transformed and are essentially used as continuous data for the fitting of an ARIMA model. The method is denoted by *FPCA*+*ARIMA*. Compared to *FPCA*+*Poisson* and *FPCA*+*ARIMA*, our proposed framework incorporates time-dependency of observations into a Bayesian generalized linear regression framework. The MSPEs of the proposed framework with P=1 or P=2 as well as the two-stage model are shown in Table 1. According to Table 1, the optimal choice of P is 2 in Simulation 2 and should be 1 in the rest of the simulations. Also, the proposed framework can produce much lower prediction MSPEs than the two-stage models, which demonstrates the benefits of estimating the unknown parameters in a joint framework.

**FIGURE 2:**
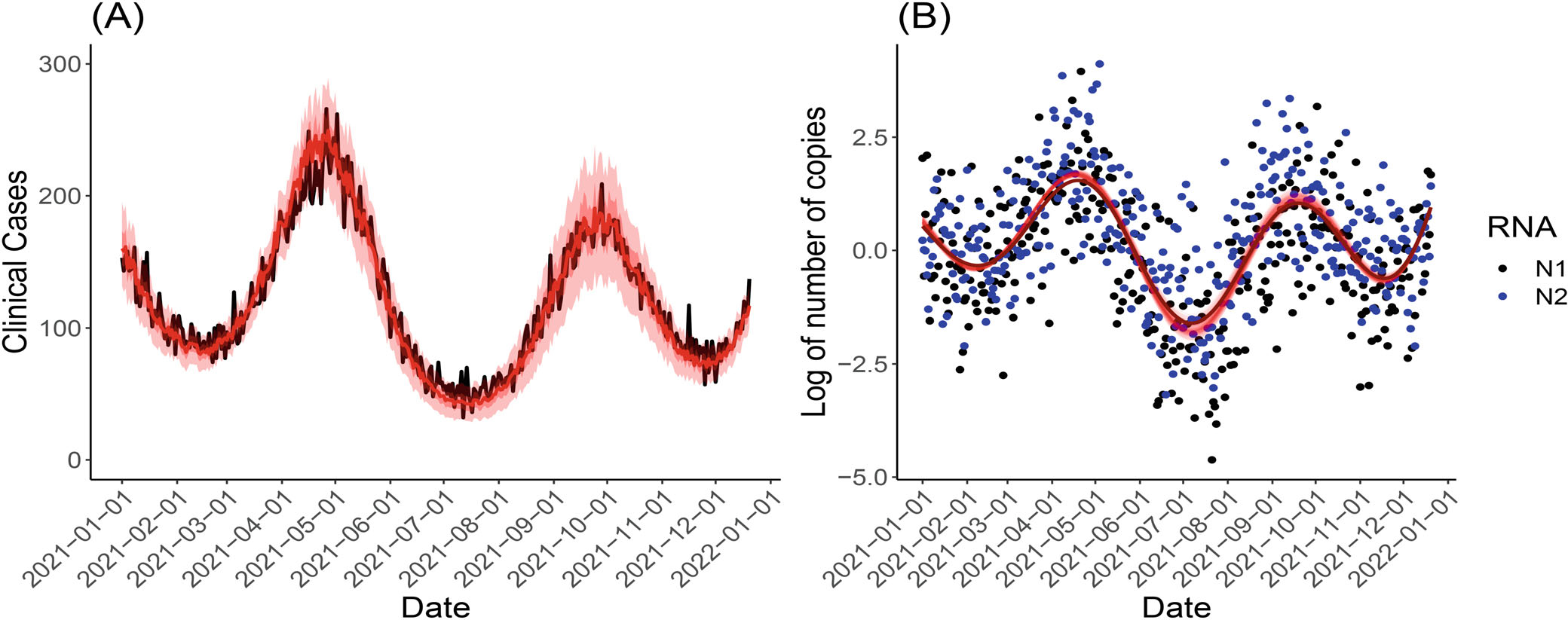
The prediction results of the proposed framework with Poisson-distributed outcome in Simulation 1. (A) The simulated numbers of cases (black line) and the mean of the MCMC sampled curves (red line). The minimum and maximum of the MCMC sampled curves are shown in the red envelope, with the 10th and the 90th percentiles indicated by the darker red inner envelope. (B) The MCMC sampled X(t) function and the simulated (log-transformed and centered) estimated number of viral genomes based on N1 and N2 gene targets. The light red curve is the MCMC sampled curves, and the dark red curve is the true X(t) function.

**FIGURE 3:**
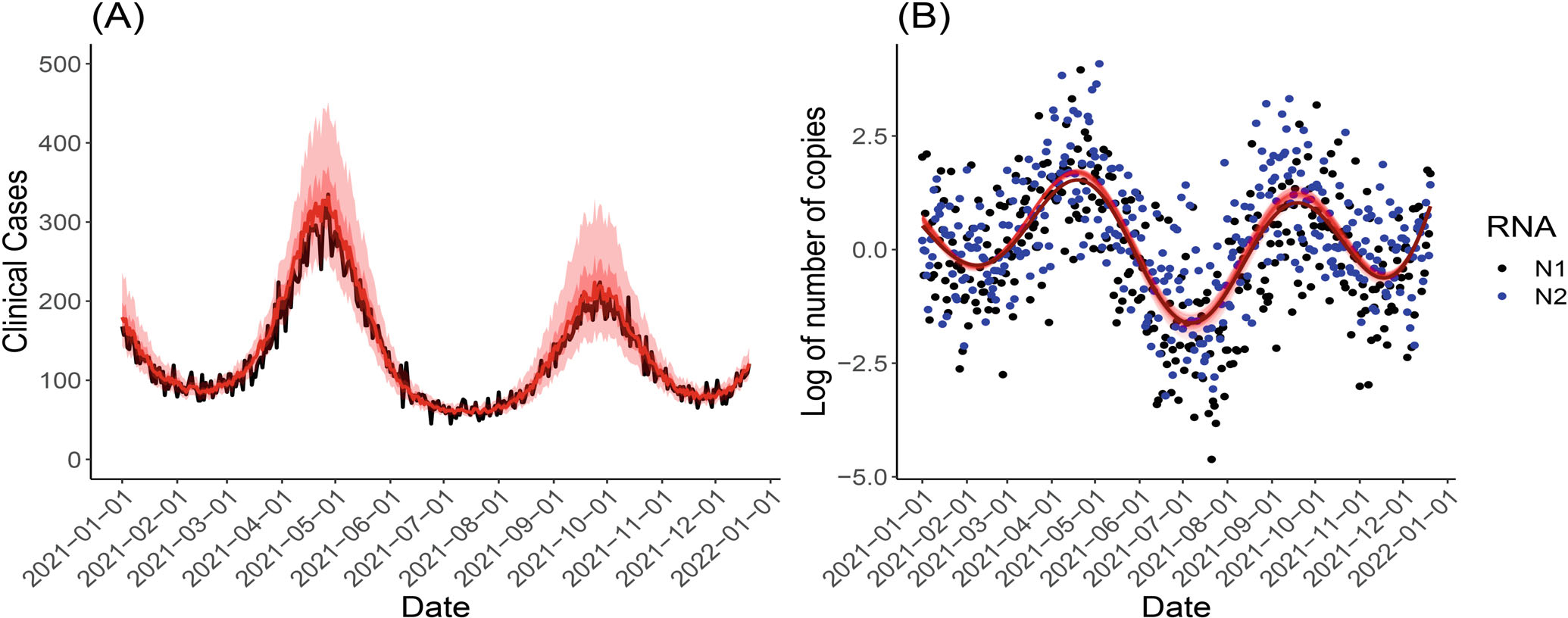
The prediction results of the proposed framework with Poisson-distributed outcome in Simulation 2. (A) The simulated numbers of cases (black line) and the mean of the MCMC sampled curves (red line). The minimum and maximum of the MCMC sampled curves are shown in the red envelope, with the 10th and the 90th percentiles indicated by the darker red inner envelope. (B) The MCMC sampled X(t) function and the simulated (log-transformed and centered) estimated number of viral genomes based on N1 and N2 gene targets. The light red curves are the MCMC sampled curves, and the dark red curve is the true X(t) function.

In Simulation 1, the effects of the WBS signals on the number of positive cases are linear, while we assume the true P is unknown to our framework. As shown in Figure 2A, the predicted number of positive cases are calculated from MCMC samples of unknown parameters as specified in Section 2.3, and the minimum and maximum and the 10th and 90th percentile (from the MCMC samples) of the daily predicted case numbers are shown in red shaded envelopes. The predicted case numbers closely track the actual simulated case numbers, which mostly fall within the lower and upper bounds of the predicted values. In Figure 2B, the simulated WBS observations for N1 and N2 gene assays are shown in black and blue dots, and the true WBS function before adding noises is shown in the dark red curve and is unknown to the proposed framework. The MCMC samples of WBS smooth signals (ie, $\overset{ˆ}{X}(t)$) are shown in light red curves of Figure 2B, which demonstrates high accuracy of unveiling the true WBS trend even when the observed WBS signals are very noisy. Note that the prediction results in Figures 2 and 3 are obtained from the proposed framework with a Poisson-distributed outcome.

Compared to Simulation 1, Simulation 2 uses the same underlying WBS function but has higher numbers of (simulated) positive cases. That is because the effects of the WBS signals on the number of positive cases are larger compared to Simulation 1. Predicted case numbers can still track the actual simulated case numbers very closely. The distributions of posterior estimates of the regression coefficients $\beta_{1}$ in Simulation 1 and $\beta_{1}$ and $\beta_{2}$ in Simulation 2 are shown in Supplementary Figure S5. True values of $\beta_{1}$ and $\beta_{2}$ belong to their 95% Bayesian credible intervals. In Simulation 3, the WBS function and the number of people tested (ie, the offset term O(t)) are obtained from the real Calgary data. The overall trend of the simulated positive cases is very similar to the actual pandemic trend observed in Calgary in the year 2021 (see Figure 1), especially for the third and fourth wave of cases (see Supplementary Figure S1). The predicted cases show high accuracy in tracking the simulated number of cases. In Simulation 4, the simulated WBS observations are longitudinal and contain a considerably large proportion of missing values. Supplementary Figure S2 shows that the predicted case numbers can still track the simulated case numbers very closely. The MCMC sampled WBS signal function still has a high accuracy of discovering the true wastewater dynamic. In Simulation 5, the number of people tested in the prediction period is treated as unknown to the proposed framework but known in the training period. Therefore, we report here the positivity rate in the prediction period estimated as $\overset{ˆ}{Y}(t)/O(t)$, where O(t) is fixed as a constant. As shown in Supplementary Figure S3, the predicted positivity rates form smooth curves as the offset term is a constant, and the simulated positivity rates (ie, simulated number of positive cases/simulated number of people tested) in the black line mostly fall within the lower and upper bounds of the MCMC samples. In Simulation 6, the simulated numbers of positive cases have larger variances compared to Simulation 1 because they are simulated from a NB distribution. As shown in Supplementary Figure S4, the posterior samples of predictions generated from the proposed framework with a NB-distributed outcome track the actual simulated cases very closely. As shown in Table 1, the proposed framework with Poisson-distributed outcome and the NB-distributed outcome have comparable results in the simulation designs.

### 3.2 | Predicting positive cases and positivity rates using observed Calgary data

Figure 1 shows the amalgamation of wastewater and clinical data in Calgary, Canada between June 2020 and January 2022. Except for the first wave of the COVID-19 pandemic, the WBS signals and clinical data of pre-Omicron pandemic waves are captured in this dataset. If we assume that the number of people tested is unknown as in practice, we will predict only positivity rates from longitudinal WBS signals. Otherwise, we will predict the number of new positive cases (as well as positivity rates) using the proposed framework with a Poisson-distributed and a NB-distributed outcome. The vaccination rate of the population (ie, the percentage of the population who has received at least one dose of government-approved COVID-19 vaccines) has been included as a covariate. As the vaccination program started in December 2020, we extended the length of the training period compared to the simulation studies so that the effect of the vaccination rate could be captured by the predictive model. To demonstrate the prediction capabilities, the predictive model is built on WBS and clinical data from June 2020 to March 2021, and the prediction period is from April 2021 to January 2022.

We aim to perform daily predictions for the number of new positive cases in the city of Calgary, and the predictions are made from leading indicators in WBS signals and one vaccination covariate one week prior (ie, the time lag in the functional regression is δ=7). As mentioned above, the FPCA process is used to interpolate and smooth the noisy and irregularly observed number of WBS signals in wastewater samples to estimate the true WBS function. To explain at least 99% of the variation in the observed WBS signals, the FPCA process keeps two eigenfunctions (ie, L=2). Also, to determine the order of effects P in Equation (3), we perform residual diagnostics for models with different *P* values using the posterior means of the residuals calculated as $Y(t)-\overset{ˆ}{Y}(t)$, where $\overset{ˆ}{Y}(t)$ is calculated as the after-burn-in posterior samples of $\overset{ˆ}{\lambda }(t)$ as detailed in Section 2.4, and the use of posterior means mimics the use of point estimates to calculate residuals in frequentist regression methods.^42^ As shown in Figure S6 of the Supplementary Information, the residuals of the proposed regression with P=1 show strong evidence of nonlinear trends, and the homogeneity of residuals has been significantly improved when the second-order effect is included in the proposed regression. Also, the model with P=2 has an average MSPE of 5522.73 for the prediction period, while the model with P=1 has an average MSPE of 6446.99. The MSPE here is calculated in the same way as in the simulation studies by comparing each after-burn-in prediction curve with the actual number of cases as ${∥Y_{T\times 1}-{\overset{ˆ}{Y}}_{T\times 1}∥}_{2}^{2}/T$. Therefore, the parameter P in Equation (3) is set to be 2. Regarding the vaccination covariate, it is not significantly associated with new positive cases after accounting for the effects of WBS signals. The posterior estimates of regression coefficients (including that of vaccination covariate: $\beta_{1}^{c}$) in the Calgary study are shown in Figure S11 of the Supplementary Information and $\beta_{1}^{c}$ has a 95% credible interval of (−1.59,1.18), which shows that 0 is not an extreme value of posterior samples of the effect of vaccination rate on RNA viral shedding.

**FIGURE 4:**
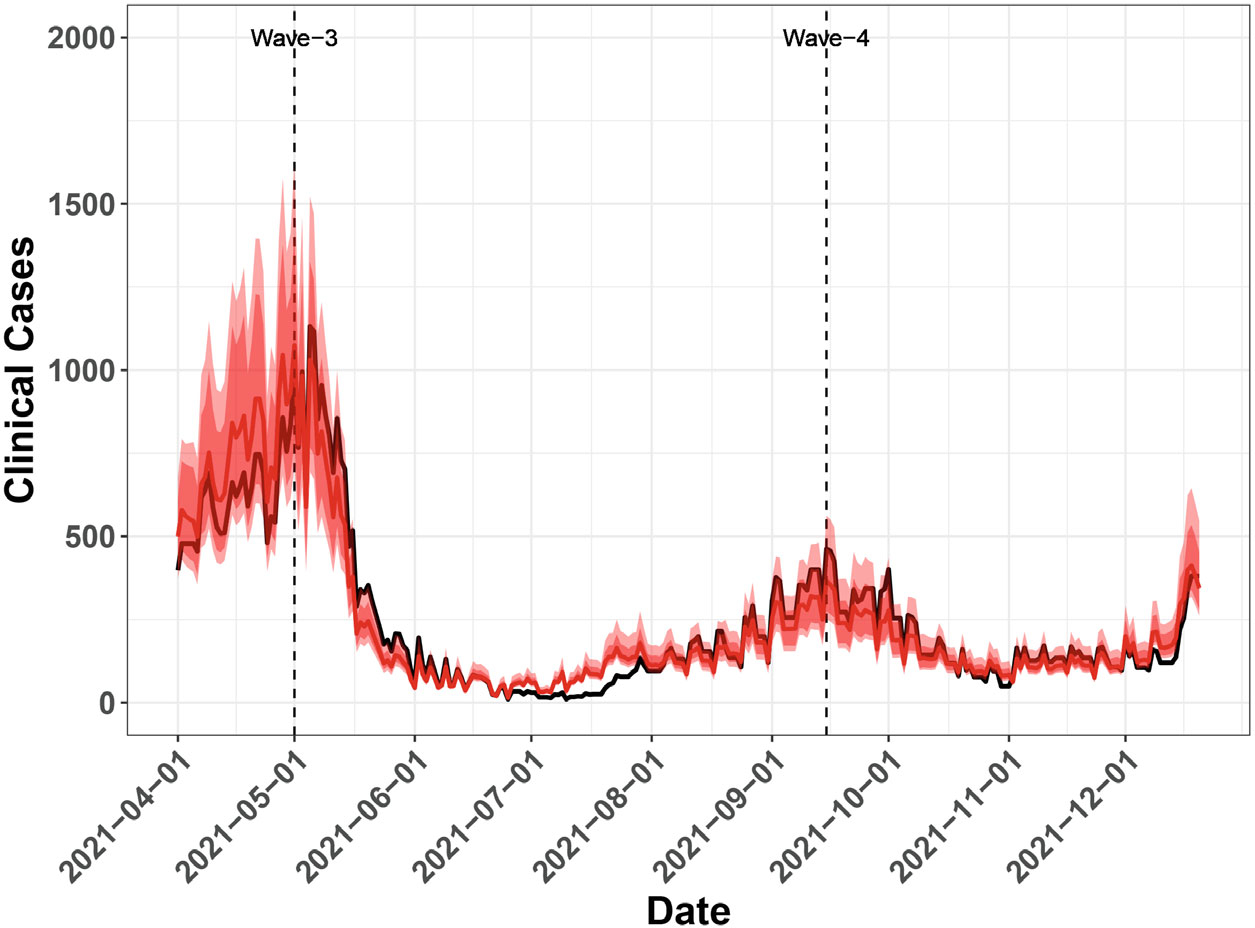
The posterior mean of the MCMC sampled curves (red line) and actual number of cases (black line). The minimum and maximum of the MCMC sampled curves are shown in the lighter red envelope, and the 5th and the 95th percentile of the sampled curves are shown in the darker red envelope.

**FIGURE 5:**
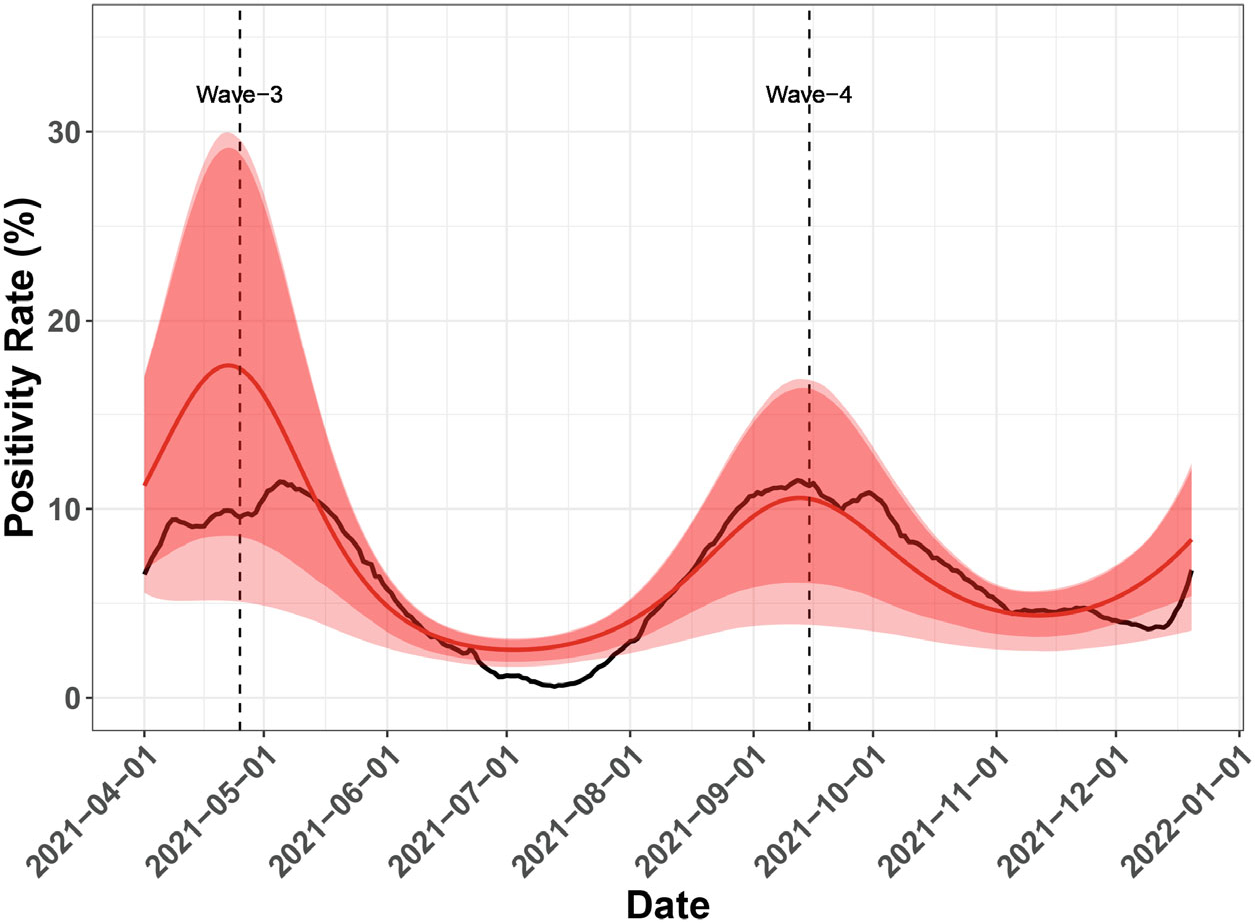
The posterior mean of the MCMC sampled curves (red line) and actual test positivity rate values (black line). The minimum and maximum of the MCMC sampled curves are shown in the lighter red envelope, and the 5th and the 95th percentile of the sampled curves are shown in the darker red envelope.

Figure 4 shows the predicted number of positive cases calculated from the MCMC samples of unknown parameters as specified in Section 2.4. Compared to the actual number of reported daily cases, the predicted numbers closely track the actual trend of positive cases with a reasonable scale of variation. If the number of people tested is unknown as is common in practice, the positivity rate will be computed and estimated from Equation (6) by setting O(t)≡1. We run 50 000 MCMC iterations with 25 000 burn-in iterations to fit our approach. The convergence of important parameters is shown in Supplementary Information Figure S7. The convergence of MCMC chains is monitored by looking at the estimated potential scale reduction factor (PSRF) from Gelman and Rubin’s convergence diagnostic^43,44^ as a function of MCMC iterations. Figure S7 shows that the PSRF values of all parameters get very close to 1 after burn-in iterations, which indicates good convergence. As shown in Figure 5, the predicted positivity rates are much smoother compared to the predicted positive cases, mostly because rates are between 0 and 1. As expected, the predicted positivity rates still closely track the actual trend of positivity rates. We perform additional Bayesian model diagnostics analyses such as the consistency of predictions in Section C of the Supplementary Information. The consistency of predictions is measured by the Bayesian *P*-value,^42^ which evaluates how well the distribution of posterior samples of predictions covers the actual outcome value. The Bayesian *P*-value results are shown in Supplementary Figures S8 and S9 for predicting new positive cases and positivity rates, respectively.

## 4 | DISCUSSION

According to Detsky and Bogoch,^45^ Canada’s third wave of COVID-19 infections occurred between March 2021 and August 2021. Data from Calgary reflect this trend but with a shorter third wave than the national average. The number of weekly confirmed COVID-19 cases peaked in April 2021 in Canada,^45^ which was consistent with our findings in wastewater RNA concentrations and the number of positive cases in Calgary. The correlation between WBS signals is expected to be weaker during waves of the Omicron variant, with clinical testing efforts being scaled down across Canada.^45^ This makes accurate interpretation of WBS signals critical for appropriate public health decision making and would be an indicator for disease outbreak monitoring. Also, Li et al^46^ discussed the number of positive cases required in the population to detect SARS-CoV-2 RNA in wastewater. The proposed Bayesian framework can account for the variation in predictions, which may be helpful when the WBS signals are less reliable.

This article proposes a joint Bayesian framework that simultaneously models WBS signals and links positive cases with WBS signal trends through a functional regression with Poisson-distributed response to predict daily positive COVID-19 cases. To link the clinical data and WBS observations with different degrees of temporal granularity, state-of-the-art functional data analysis techniques have been utilized, especially the FPCA process. The great potential of applying functional data analysis techniques to longitudinal wastewater monitoring (ie, intermittent wastewater sampling) data has been demonstrated in this work. Also, the adoption of a functional regression framework and the FPCA process have seamlessly integrated the clinical and WBS observations into one predictive model, regardless of their differences in frequencies and sparseness. Other literature proposed to use linear regression^9^ and/or nonlinear regressions such as neural networks^11^ to predict COVID-19 cases, but, to the best of our knowledge, our proposed method pioneers the integration of imputation of sparse WBS observations and prediction of clinical cases into a joint Bayesian framework using functional data analysis techniques. Besides imputing sparse wastewater observations, the FPCA process also removes measurement errors in wastewater observations and smoothes the signal function. The measurement error (or bias) can be caused by known and unknown factors. We perform normalization for known factors (eg, water flow, temperature) and smoothing for unknown factors on raw wastewater observations. However, an important question is whether smoothing can also remove useful information. Our current approach is to let the FPCA process retain at least 99% of variation in original observations. In future studies, we can further investigate the measurement errors in wastewater observations, which is also an active research field of functional data analysis.^47^

In addition, the proposed framework requires little fine-tunings compared to existing methods. For example, McMahan et al^21^ proposed to predict the number of infected individuals from WBS data using a system of differential equations. The underlying assumption made by McMahan et al^21^ is that the rate of change in the proportion of the population who are infectious is a factor of the proportion of the population who are exposed, where the factor is assumed to be 0.2 in the manuscript. Our approach has fewer parameters with unknown parameters directly estimated from the observed data. Also, the work of hyperparameter tuning (mostly involving P) is minimal as demonstrated in the simulation studies and real data analysis. This advantage is important because the predictions and biological interpretations should be largely consistent regardless of the choices of hyperparameters.

In this article, the proposed framework is developed to predict both the case numbers and the positivity rates. This framework has the potential to infer ICU rates and infection fatality rates (IFR; the number of COVID-19 related deaths divided by the number of positive cases).^48^ The number of positive cases is treated as the denominator (ie, offset) if predicting IFR is the goal. A Bayesian model can better account for uncertainty in both the number of deaths and unknown parameters.^48^

As of early 2022, there are nearly 500 million confirmed COVID-19 cases worldwide.^48^ WBS might be the only option for providing rapid, inexpensive mass pandemic surveys.^12^ There are also other emerging public health challenges such as new pandemics and drug abuse,^49^ which all require better understanding and modeling of wastewater surveillance data. This study aims to contribute to wastewater surveillance research from two directions. The first direction is the high quality of the original wastewater data collected from Calgary and surrounding areas. The separate wastewater and stormwater collection systems enable more accurate surveillance of wastewater-based epidemiology, which can be demonstrated in the strong correlation between WBS observations and clinical outcomes. The Calgary study proves the WBS a powerful tool for research groups and government agencies to track community-wide infections, not only for the COVID-19 pandemic but also for other infectious diseases. In this article, the Calgary study serves as a proof-of-concept study for the performances of the proposed framework. The proposed framework can be applied to similar wastewater studies with recorded positive cases in certain communities/regions and intermittent wastewater samplings. The proposed framework also has the potential to predict hospitalizations and ICU admissions from wastewater surveillance data. We demonstrate this potential through an additional study of predicting ICU admissions in Calgary as detailed in Section D of the Supplementary Information. The second direction is the novel Bayesian framework proposed to bridge the gap between state-of-the-art statistical methodology and real-world epidemiology data because there are many uncertainties (eg, frequency of observations, statistical errors) to account for in such a large-scale study. The proposed framework is robust enough to handle many of these challenges facing the WBS modeling. It is also demonstrated in the simulations that a joint Bayesian framework significantly outperforms a two-stage method.

## Supplementary Material

Supplementary material

## Figures and Tables

**TABLE 1:** Comparison of different methods on simulated data.

Simulation	Poisson outcome	NB outcome	FPCA + Poisson	FPCA + ARIMA

1	223.85 (*P* = 1; 1.86)	245.62 (*P* = 1; 2.43)	406.36	597.60
2	453.33 (*P* = 2; 6.74)	383.09 (*P* = 2; 8.02)	3308.12	3648.30
3	235.71 (*P* = 1; 1.01)	239.27 (*P* = 1; 1.35)	372.31	980.64
4	207.60 (*P* = 1; 0.38)	205.58 (*P* = 1; 0.57)	746.95	453.83
5	0.32% (*P* = 1; 0.0006%)	0.33% (*P* = 1; 0.0007%)	0.39%	0.38%
6	478.10 (*P* = 1; 2.61)	464.39 (*P* = 1; 2.56)	900.88	4700.69

*Note*: The proposed framework is fitted with a Poisson-distributed outcome and a negative binomial-distributed outcome and each outcome with *P* = 1 and *P* = 2. For (simulated) observed case counts $Y_{T\times 1}$, the MSPE (${∥Y_{T\times 1}-{\overset{ˆ}{Y}}_{T\times 1}∥}_{2}^{2}/T$) of predictions is calculated. Note that the MSPE included in this table is the lesser of the model fitted with *P* = lvs *P* = 2, with the optimal *P* in the parenthesis. The standard errors of the MSPEs for the proposed framework are also included in the parenthesis. The frequentist methods compared are FPCA plus Poisson regression and FPCA plus ARIMA. In Simulation 5, the MSPE is calculated by comparing predicted positivity rates with simulated positivity rates. For the negative binomial-distributed outcome, we let *η* = 10,100, and 1000, and choose the one with the minimum MSPE.

## Data Availability

The R code for the implementation of methods is available as an R package: https://github.com/xiaotiand/WBSpred. The wastewater data and other data types (eg, number of cases) are also available in the R package.
