# Supplementary material for: A Bayesian framework for modeling COVID-19 case numbers through longitudinal monitoring of SARS-CoV-2 RNA in wastewater
Source: Stat Med. Author manuscript; Available in PMC 2024 Jul 12. (PMC11239317; doi:10.1002/sim.10009)
Supplement: Supplementary material [file NIHMS2001778-supplement-Supplementary_material.pdf]

Supplementary Information for "A Bayesian framework for modeling COVID-19 case numbers through longitudinal monitoring of SARS-CoV-2 RNA in wastewater"

November 8, 2023

A Wastewater RT-qPCR analysis

RT-qPCR was used to quantify SARS-CoV-2-RNA in wastewater. United States Center for Disease Control (US-CDC) primers and probes were used to amplify two regions of the nucleocapsid gene (i.e., N1 and N2). Amplification reactions for SARS-CoV-2 detection were performed in triplicate following the N1 and N2 assays previously described (Acosta et al., 2021). Samples were considered positive for N1 or N2 gene targets if the threshold quantification cycle (Cq) was lower than 40 cycles (Acosta et al., 2021). Total mass flux of SARS-CoV-2 RNA concentration measured as copies per day for the city of Calgary was calculated as previously described by Acosta et al. (2022), in which the daily flow rate of each WWTP was multiplied to copies/mL of SARS-CoV-2 RNA measured in wastewater to generate the number of copies per day for each WWTP (Acosta et al., 2022). For this reason, the RNA concentration (copies per day) across three WWTPs in Calgary can be aggregated into one data point per sampling day to represent city-wide SARS-CoV-2 burden.

B Simulation results

Additional numerical analysis of the simulations are shown in Table 1 and 2. Additional simulation results are shown in Figure S1, S2, S3, S4, and S5. See Section 3 of the main text for their descriptions.

C Model diagnostics analysis of Calgary data

As shown in Figure S6, the residuals of the proposed regression with $P = 1$ shows strong evidence of non-linear trends, and the homogeneity of residuals has been significantly improved when the second-order effect is included in the proposed regression. The smooth lines in Figure S6 are fitted to the scatter plots of residuals using local polynomial regression (Cleveland and Grosse, 1991).

We also check the performance of predictions using the posterior predictive checking method described in Section 6.3 of Gelman et al. (1995). The method checks the proportion of MCMC samples of prediction values on the test data that are larger than the actual value: $P(\hat{Y}_t > Y_t)$, which can be seen as the tail-area probability of the actual value in

Table 1: Numerical analysis of simulation results. For estimated WBS function, the average MSE is calculated, and the standard errors of the averages are included in the parenthesis. The results are obtain from a Poisson-distributed outcome.

Simulation	$\|\|\boldsymbol{X}_{T\times 1} - \hat{\boldsymbol{X}}_{T\times 1}\|\|_2^2/T$
1	0.01 (8.07×10^{-5})
2	0.03 (8.47×10^{-4})
3	0.01 (1.36×10^{-5})
4	0.02 (2.80×10^{-4})
5	0.02 (2.04×10^{-4})

Table 2: Additional comparisons of different methods on simulated data. For the proposed framework fitted with the Poisson-distributed outcome and $P = 1$ and $P = 2$, the average MSPE are calculated, and the standard errors of the averages are included in the parenthesis. For other frequentist methods, the MSPE is calculated by comparing predicted case numbers with true case numbers. In Simulation 5, the MSPE is calculated by comparing predicted positivity rates with actual positivity rates.

Simulation	P = 1	P = 2
1	223.85 (1.86)	250.64 (1.71)
2	1636.26 (15.79)	453.33 (6.74)
3	235.71 (1.01)	277.83 (1.75)
4	207.60 (0.38)	229.31 (0.46)
5	0.32% (0.0006%)	0.42% (0.0025%)

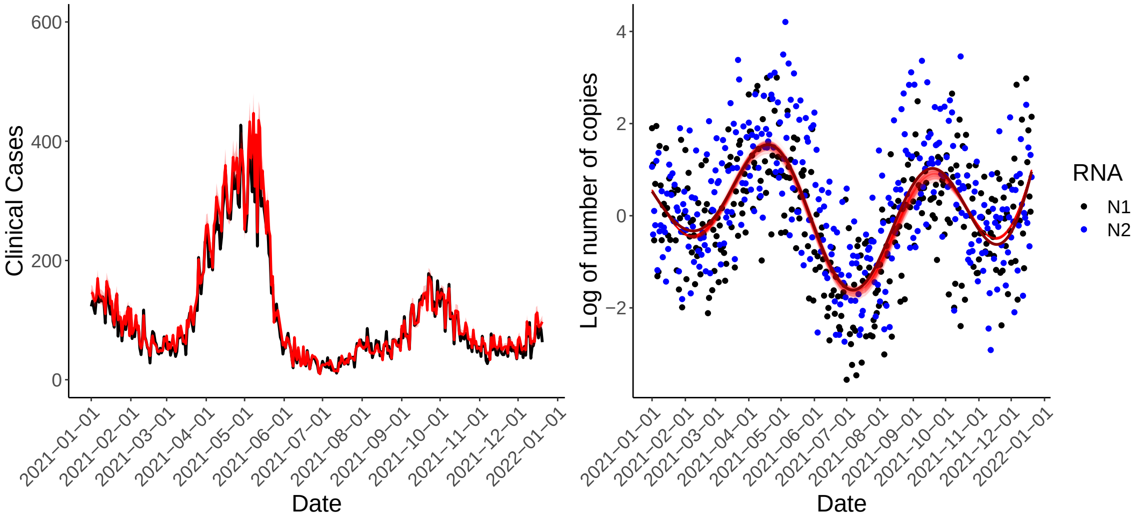

Figure S1: The prediction results of the proposed framework with Poisson-distributed outcome in Simulation 3. (A): The simulated number of cases (black line) and the mean of the MCMC sampled curves (red line). The minimum and maximum of the MCMC sampled curves are shown in red envelop, with the 10th and the 90th percentiles indicated by the darker red inner envelope. (B): The MCMC sampled $X(t)$ function and the simulated (log-transformed and centered) estimated number of viral genomes based on N1 and N2 gene targets. The light red curves are the MCMC sampled curves, and the dark red curve is the true $X(t)$ function.

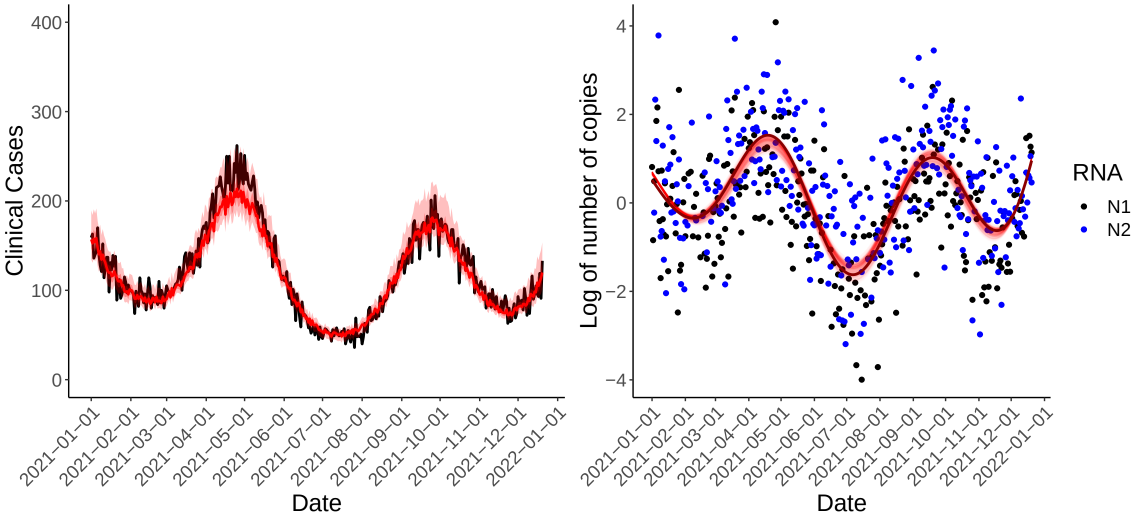

Figure S2: The prediction results of the proposed framework with Poisson-distributed outcome in Simulation 4. (A): The simulated number of cases (black line) and the mean of the MCMC sampled curves (red line). The minimum and maximum of the MCMC sampled curves are shown in red envelop, with the 10th and the 90th percentiles indicated by the darker red inner envelope. (B): The MCMC sampled $X(t)$ function and the simulated (log-transformed and centered) estimated number of viral genomes based on N1 and N2 gene targets. The light red curves are the MCMC sampled curves, and the dark red curve is the true $X(t)$ function.

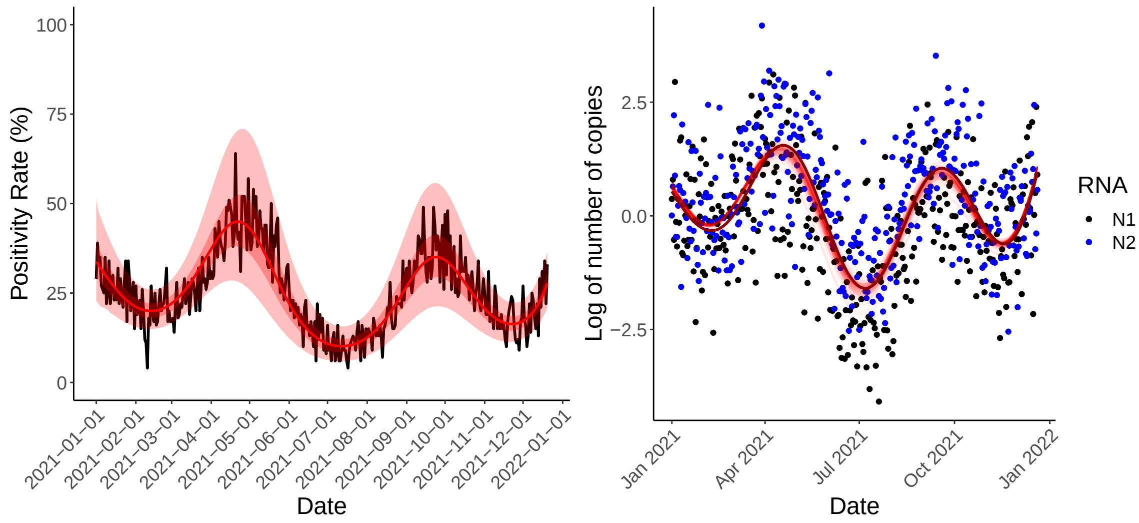

Figure S3: The prediction results of the proposed framework with Poisson-distributed outcome in Simulation 5. (A): The simulated number of cases (black line) and the mean of the MCMC sampled curves (red line). The minimum and maximum of the MCMC sampled curves are shown in red envelop, with the 10th and the 90th percentiles indicated by the darker red inner envelope. (B): The MCMC sampled $X(t)$ function and the simulated (log-transformed and centered) estimated number of viral genomes based on N1 and N2 gene targets. The light red curves are the MCMC sampled curves, and the dark red curve is the true $X(t)$ function.

the distribution of predicted values. Therefore, this proportion is also referred to as the Bayesian p-value. A Bayesian p-value that is too large or too small (e.g., beyond the 99% confidence bounds) indicates that the distribution of predictions does not cover the true value very well. In Supplementary Information Figures S8 and S9, the Bayesian p-values are calculated for the predicted positive cases and positivity rates in the prediction period. For most of the predictions, their Bayesian p-values are between the lower bound and upper bound, indicating that the actual values are not in the tail-areas of prediction distributions and the prediction performance of the proposed framework is reasonably strong. As shown in Supplementary Information Figure S10, the MCMC samples of WBS signal function $X(t)$ are compared with actual observations of N1 and N2 copies in wastewater of Calgary. The sampled $X(t)$ functions have relatively small variation and reflect the overall dynamics of WBS observations except for the observations in early July 2021 when there was a sudden drop in WBS observations and in positive cases. Due to the smoothing of trajectories in the FPCA process, the sampled $X(t)$ functions cannot drop as much as the actual observations do, which explains why the only time when the actual values fall outside the prediction bands in Figures 4 and 5 of the main text happens in early July of 2021. The distributions of posterior estimates of the regression coefficients β_1, β_2 and β_1^c are shown in Supplementary Information Figure S11. The mean of β_1's posterior estimates is 0.47, and the mean of β_2's posterior estimates is 0.03. Also, the credible interval (CI) of β_2's samples dose not cover zero, which further proves the significance of the effects of squared WBS signals on modeling case numbers.

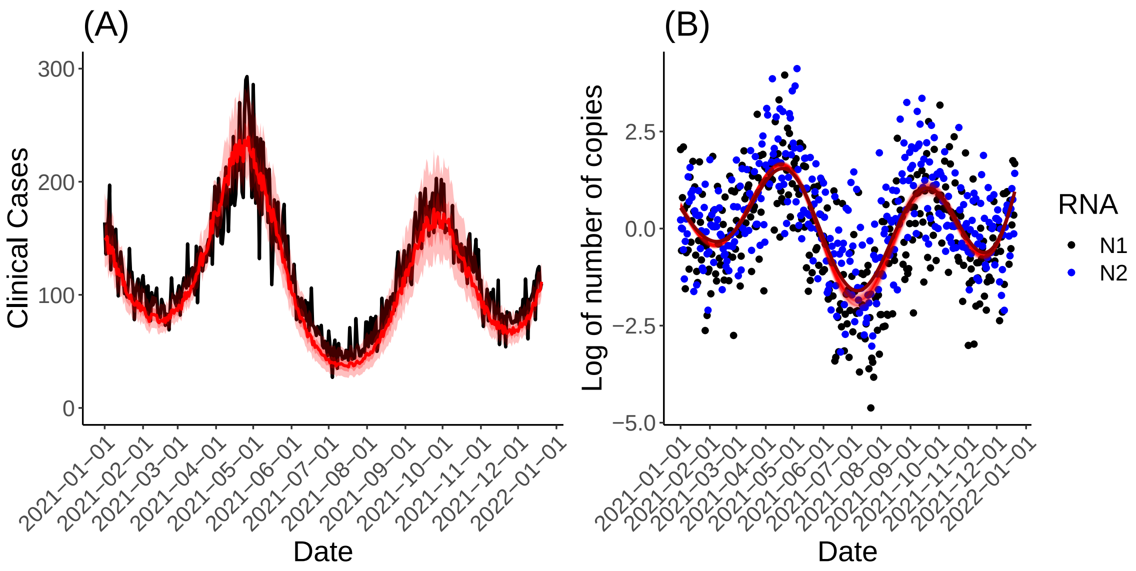

Figure S4: The prediction results of the proposed framework with Negative Binomial-distributed outcome in Simulation 6. (A): The simulated number of cases (black line) and the mean of the MCMC sampled curves (red line). The minimum and maximum of the MCMC sampled curves are shown in red envelop, with the 10th and the 90th percentiles indicated by the darker red inner envelope. (B): The MCMC sampled $X(t)$ function and the simulated (log-transformed and centered) estimated number of viral genomes based on N1 and N2 gene targets. The light red curves are the MCMC sampled curves, and the dark red curve is the true $X(t)$ function.

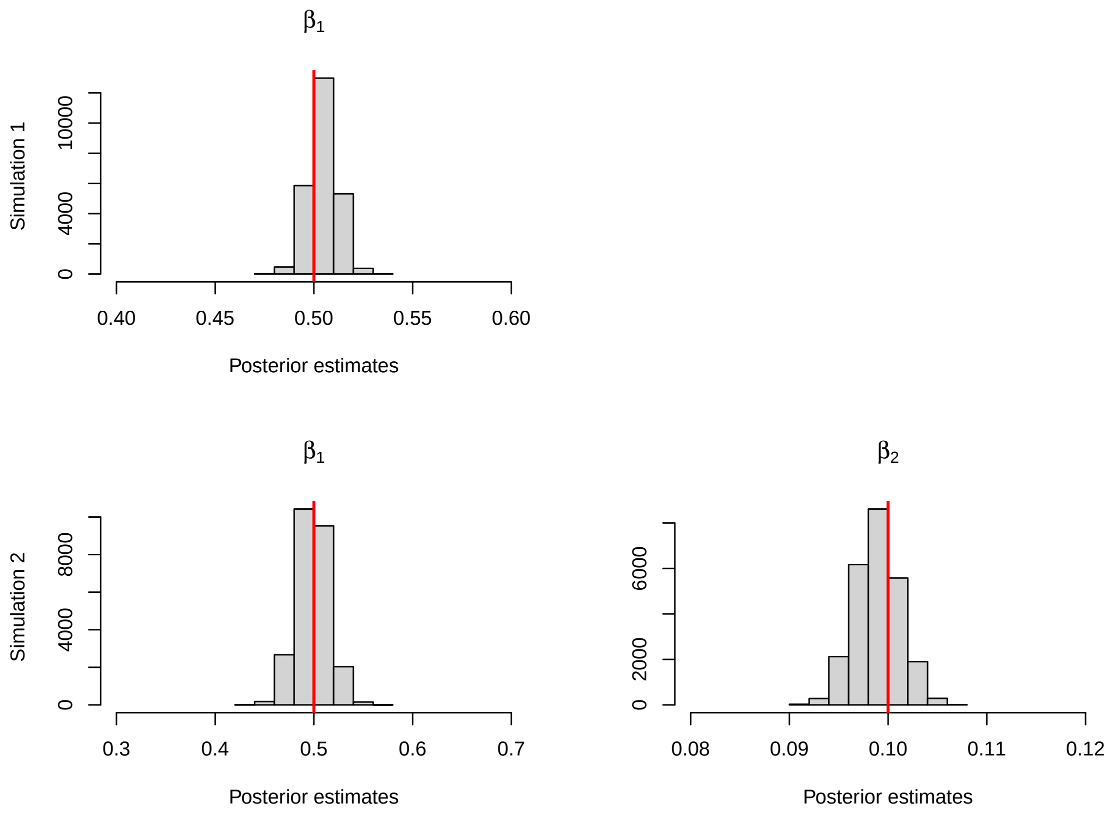

Figure S5: The distributions of posterior estimates of the regression coefficients β_1 and β_2 in Simulations 1 and 2. The red line shows the real values of regression coefficients. The y-axis shows the number of posterior samples in the corresponding interval.

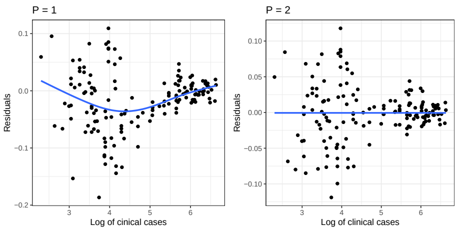

Figure S6: Residual diagnostics plots with log of positive cases vs. residuals of Poisson regressions. The smooth line is fitted using the *ggplot2* package in R. The residual of the model with $P = 1$ (left plot) shows non-linear trend of residuals. The residual of the model with $P = 2$ (right plot) shows great improvement of residual homogeneity compared to the left plot.

D Predicting intensive care unit (ICU) admissions in the city of Calgary

As an extension of our Calgary study in the main text, we implement daily predictions for the number of ICU admissions with a positive COVID-19 test (referred to as the ICU admissions later) in the city of Calgary. The offset term used here is the number of hospitalized patients with a positive COVID-19 test (referred to as the hospitalized cases later) in Calgry. Note that the hospitalized cases include all admitted patients with a positive (and mandatory) COVID-19 test, but COVID-19 may not be the primary reason of hospitalization. Following the Calgary study on the number of positive cases, the predictive model is built on WBS and clinical data from June, 2020 to March, 2021, and the prediction period is from April, 2021 to January, 2022. The proposed framwork is fitted with a Poisson-distributed outcome and the first and second power of WBS signals (i.e., $P = 2$).

Figure S12 shows the predicted number of ICU admissions calculated from the MCMC samples of unknown parameters as specified in Section 2.4 of the main text. Compared to the actual number of reported daily cases, the predicted numbers can mostly track the actual trend of ICU admissions with reasonable scale of variation. However, the associations between ICU admissions and city WBS signals may not be as strong as those between positive cases and city WBS signals because all infected residents can shed viruses. The proposed framework can predict positive cases more accurately as shown in Section 3.2 of the main text than predicting ICU admissions. To improve predictions, a possible solution is to sample wastewater from the hospital community sewer system instead of city wastewater plants.

References

Acosta, N., Bautista, M. A., Hollman, J., McCalder, J., Beaudet, A. B., Man, L., Waddell, B. J., Chen, J., Li, C., Kuzma, D. et al. (2021) A multicenter study investigating SARS-CoV-2 in tertiary-care hospital wastewater. Viral burden correlates with increasing hospitalized cases as well as hospital-associated transmissions and outbreaks. *Water Research*, 117369.

Acosta, N., Bautista, M. A., Waddell, B. J., McCalder, J., Beaudet, A. B., Man, L., Pradhan, P., Sedaghat, N., Papparis, C., Bacanu, A. et al. (2022) Longitudinal SARS-CoV-2

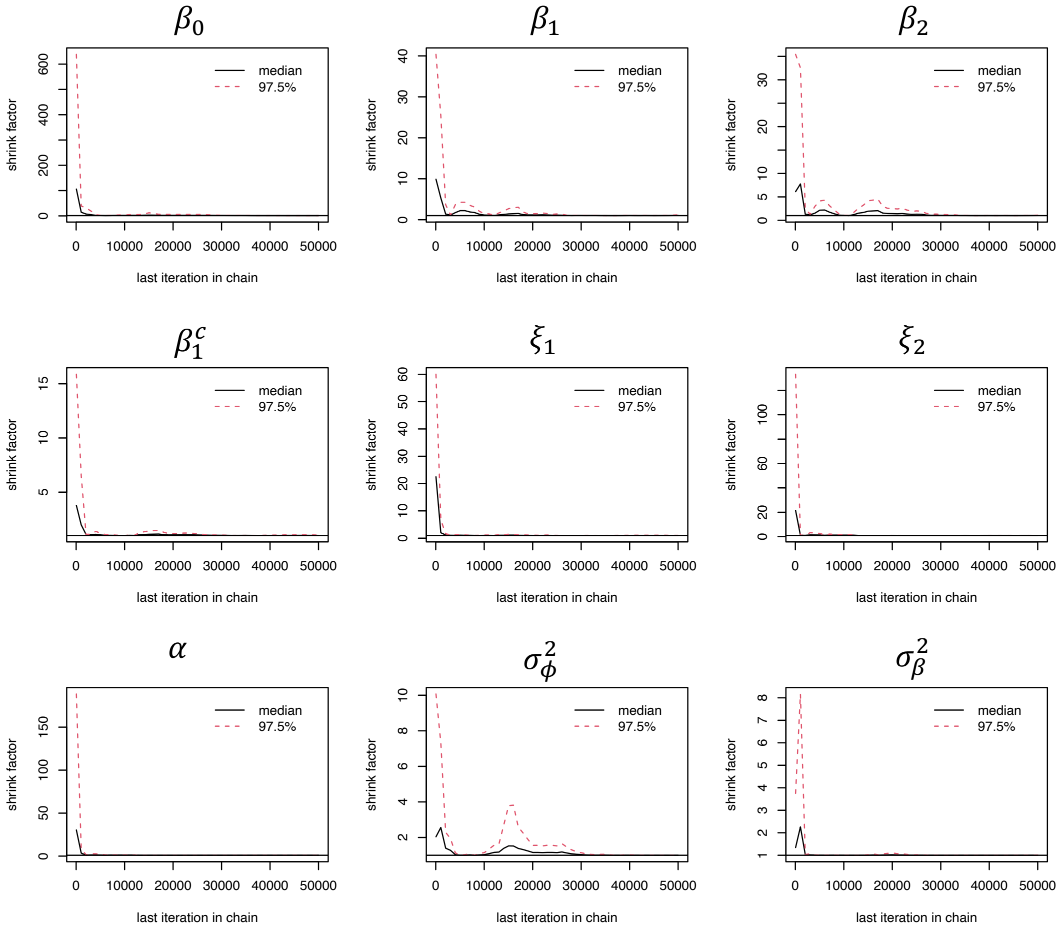

Figure S7: Gelman-Rubin-Brooks plot of the training model in Calgary. The plot assess the convergence of MCMC chains. A PSRF value close to 1 indicates good convergence of random parameter.

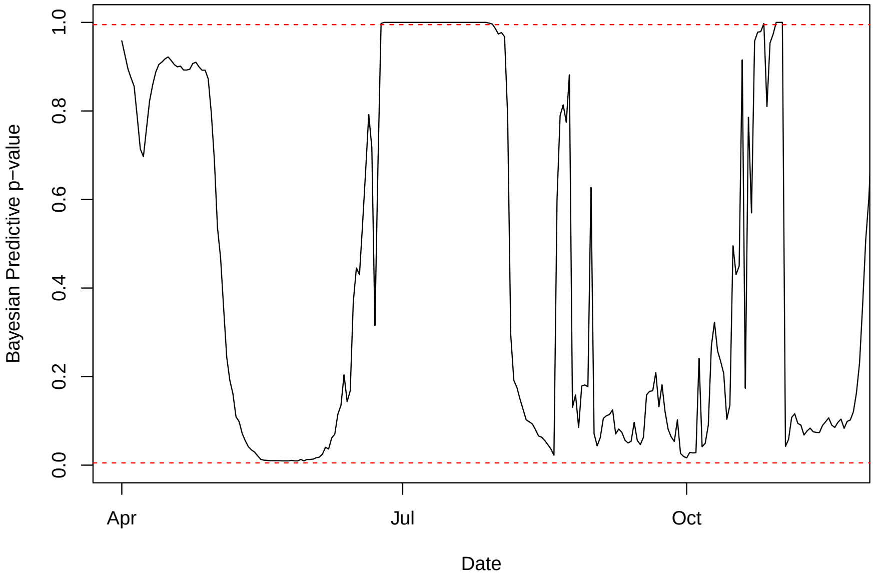

Figure S8: The results for posterior predictive checking for the model predicting case numbers, which is also known as the Bayesian predictive p-value. The value is calculated as the proportion of predicted values that are greater than the actual value, and a value that is close to 0 or 1 indicates poor prediction. The total tail-area probability (beyond the red lines) is 1%.

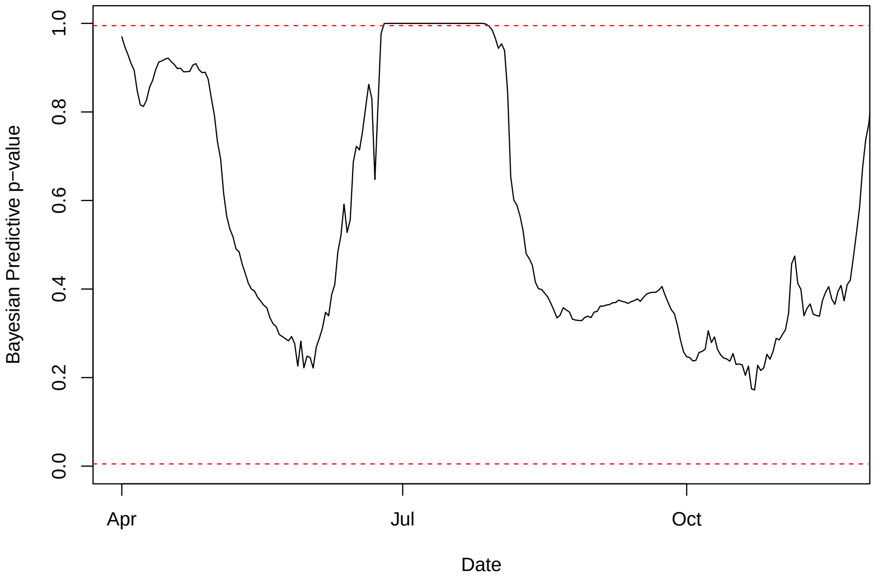

Figure S9: The results for posterior predictive checking for the model predicting positivity rates, which is also known as the Bayesian predictive p-value. The value is calculated as the proportion of predicted values that are greater than the actual value, and a value that is close to 0 or 1 indicates poor prediction. The total tail-area probability (beyond the red lines) is 1%.

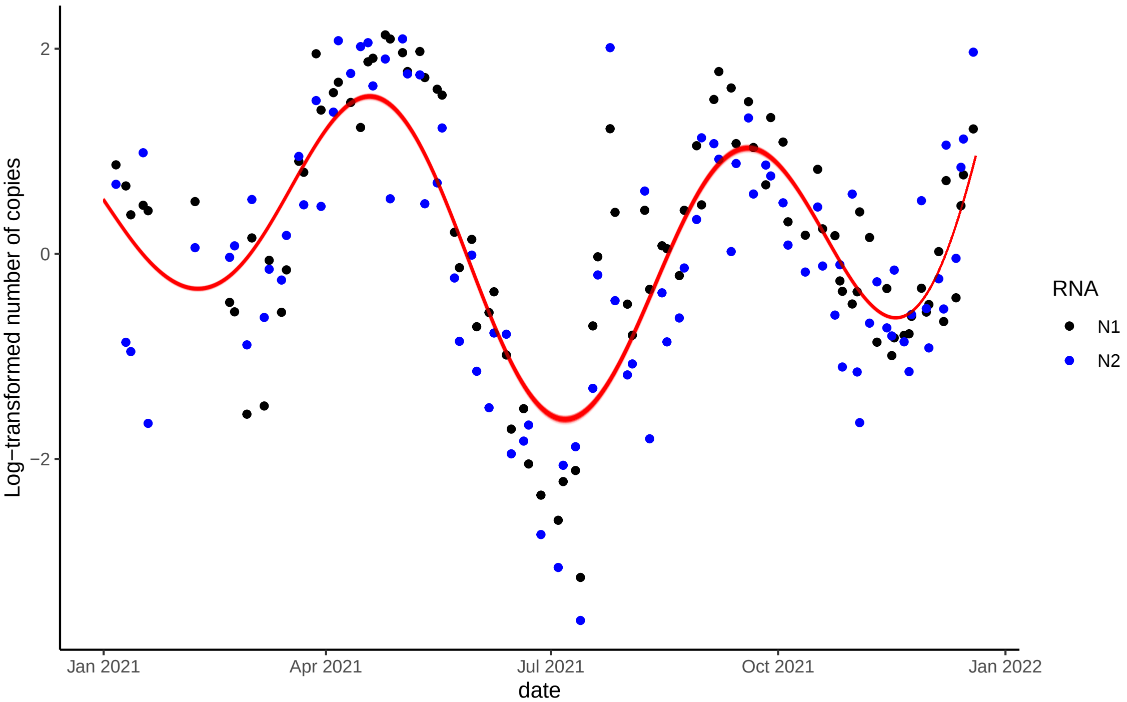

Figure S10: The MCMC sampled $X(t)$ function and the observed (log-transformed and centered) numbers of copies of N1 and N2 viruses in Calgary. The sampled $X(t)$ functions are generated from the proposed integrative framework.

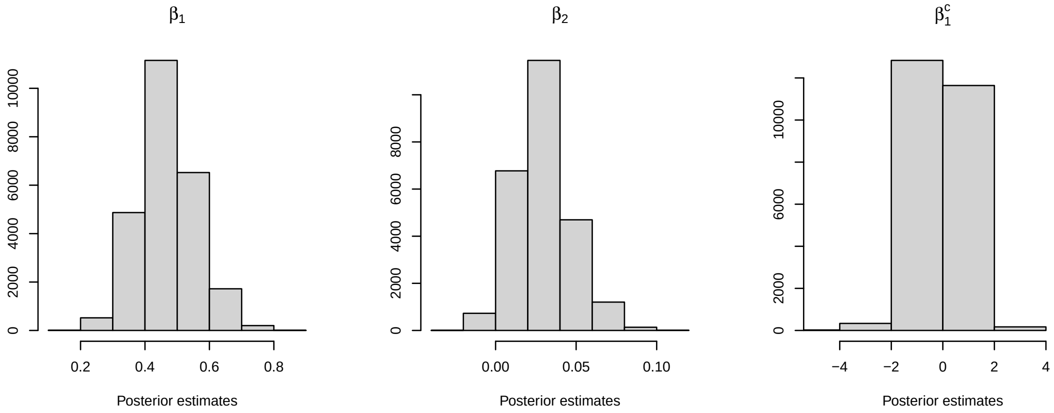

Figure S11: The distributions of posterior estimates of the regression coefficients β_1, β_2 and β_1^c. The y-axis shows the number of after-burn-in posterior samples in the corresponding interval.

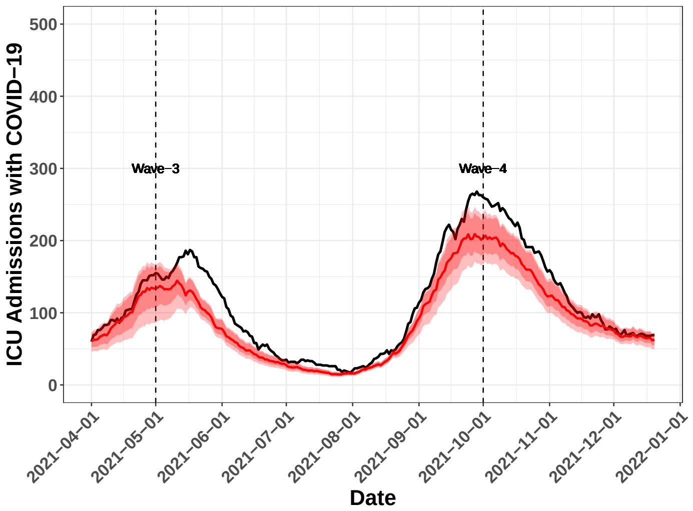

Figure S12: The posterior mean of the MCMC sampled curves (red line) and actual number of cases (black line). The minimum and maximum of the MCMC sampled curves are shown in the lighter red envelope, and the 5th and the 95th percentile of the sampled curves are shown in darker red envelope.

RNA wastewater monitoring across a range of scales correlates with total and regional COVID-19 burden in a well-defined urban population. *Water Research*, 118611.

Cleveland, W. S. and Grosse, E. (1991) Computational methods for local regression. *Statistics and Computing*, **1**, 47–62.

Gelman, A., Carlin, J. B., Stern, H. S. and Rubin, D. B. (1995) *Bayesian Data Analysis*. No. 143-153. Chapman and Hall/CRC.

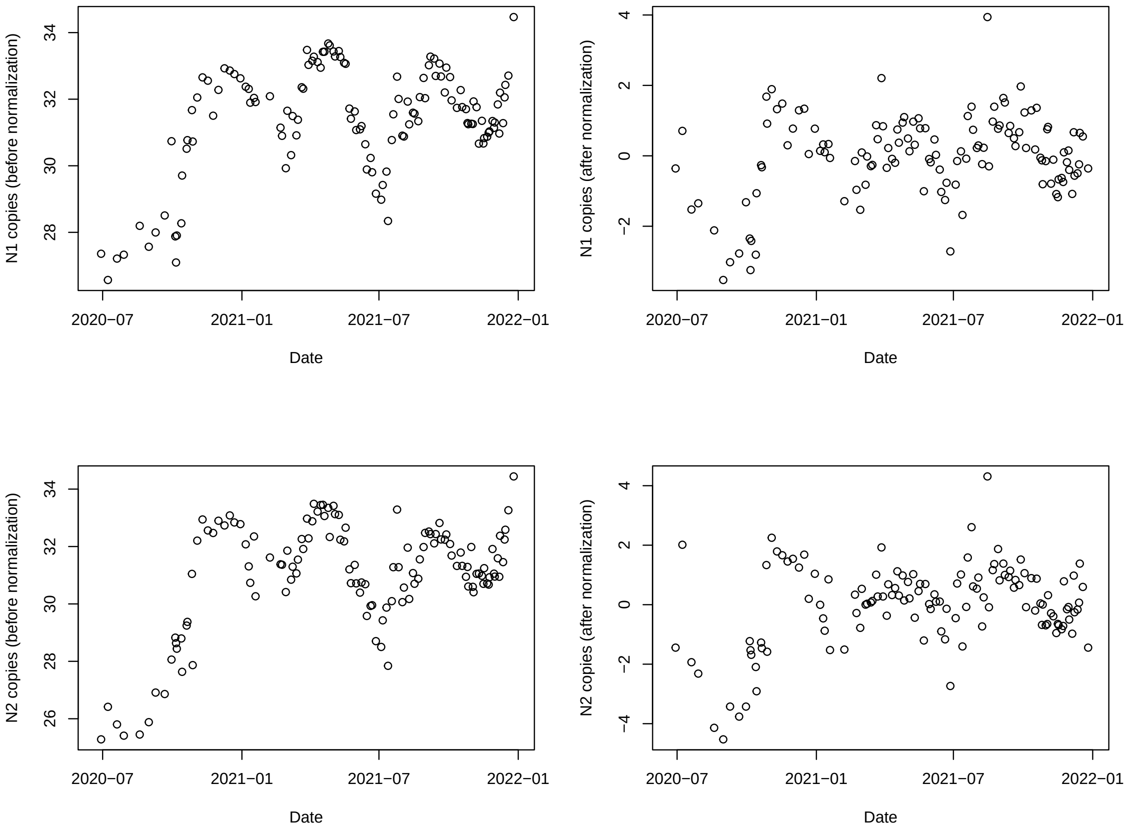

Figure S13: The log-transformed numbers of N1 copies (first row) and N2 copies (second row) in all Calgary wastewater plants before and after normalization.
